# Structural integrity assessment of Inconel 617/P92 steel dissimilar welds for different groove geometry

**DOI:** 10.1038/s41598-023-35136-1

**Published:** 2023-05-17

**Authors:** Amit Kumar, Chandan Pandey

**Affiliations:** Mechanical Department, Indian Institute of Technology Jodhpur, Karwar, Rajasthan India

**Keywords:** Engineering, Materials science

## Abstract

The work is focused on examining the effect of the weld groove geometry on microstructure, mechanical behaviour, residual stresses and distortion of Alloy 617/P92 steel dissimilar metal weld (DMW) joints. Manual multi-pass tungsten inert gas welding with ERNiCrCoMo-1 filler was employed to fabricate the DMW for two different groove designs: Narrow V groove (NVG) and Double V groove (NVG). The microstructural examination suggested a heterogeneous microstructure evolution at the interface of the P92 steel and ERNiCrCoMo-1 weld, including the macrosegregation and element diffusion near the interface. The interface structure included the beach parallel to the fusion boundary at the P92 steel side, the peninsula connected to the fusion boundary and the island within the weld metal and partially melted zone along Alloy 617 fusion boundary. An uneven distribution of beach, peninsula and island structures along the fusion boundary of P92 steel was confirmed from optical and SEM images of interfaces. The major diffusion of the Fe from P92 steel to ERNiCrCoMo-1 weld and Cr, Co, Mo, and Ni from ERNiCrCoMo-1 weld to P92 steel were witnessed from SEM/EDS and EMPA map. The Mo-rich M_6_C and Cr-rich M_23_C_6_ phases were detected in inter-dendritic areas of the weld metal using the weld’s SEM/EDS, XRD and EPMA study, which formed due to the rejection of Mo from the core to inter-dendritic locations during solidification. The other phases detected in the ERNiCrCoMo-1 weld were Ni_3_(Al, Ti), Ti(C, N), Cr_7_C_3_ and Mo_2_C. A variation in the microstructure of weld metal from top to root and also along the transverse direction in terms of composition and dendritic structure and also due to the composition gradient between dendrite core and inter-dendritic areas, a significant variation in hardness of weld metal was observed from both top to root and also in the transverse direction. The peak hardness was measured in CGHAZ of P92 while the minimum was in ICHAZ of P92 steel. Tensile test studies of both NVG and DVG welds joint demonstrated that failure occurred at P92 steel in both, room-temperature and high-temperature tensile tests and ensured the welded joint’s applicability for advanced ultra-supercritical applications. However, the strength of the welded joint for both types of joints was measured as lower than the strength of the base metals. In Charpy impact testing of NVG and DVG welded joints, specimens failed in two parts with a small amount of plastic deformation and impact energy of 99 ± 4 J for the NVG welds joint and 91 ± 3 J for the DVG welded joint. The welded joint met the criteria for boiler applications in terms of impact energy (minimum 42 J as per European Standard EN ISO15614-1:2017 and 80 J as per fast breeder reactor application). In terms of microstructural and mechanical properties, both welded joints are acceptable. However, the DVG welded joint showed minimum distortion and residual stresses compared to the NVG welded joint.

## Introduction

Many countries have accelerated the construction of advanced ultra-supercritical (AUSC) thermal power units with high steam temperatures and pressure in order to achieve high energy efficiency with minimal carbon footprints^1–3^. The AUSC technology is one of the recent technologies for next-generation power units, which have great potential for energy conservation and can be commercialized with efficiency up to ~ 50%^4^. The AUSC units can reduce CO_2_ emission by 22% as compared to conventional power units^5^. In the 1960s, the first USC unit (Eddystone power plant) was constructed to operate at a temperature of 650 °C and pressure of 34.5 MPa. After that, a rapid increase in USC technology has been observed in Europe, Japan and China. The maximum thermal efficiency for Laiwu 100 MW USC power units, operating at a temperature of 600 °C with a double reheater, was approximately 48.12%. After the successful design and operation of the 600 °C USC power plants and the breakthrough in the advanced material operating at high temperatures and pressures, a target of developing AUSC units was another agenda in 2001 like 700 CE programme (European), A-USC programme 760 (American), and A-USC programme (Japanese)^2^. As compared to 600 °C USC technology, AUSC technology shows an increase in temperature and pressure by 100 °C and 10 MPa, respectively. USC power plant operates at a temperature of about 600 °C/620 °C with a steam pressure of 25–30 MPa while AUSC power plants operate at a temperature of approximately 700 °C/760 °C with a steam pressure of about 35 MPa^6^. The increase in temperature and pressure of working fluid demands a higher operating temperature of materials for safe operation. Most widely two types of material are used for AUSC power plants, firstly high-temperature creeps resistant ferritic/martensitic steel also known as 9%Cr–Mo steel like P91/P92 steel and secondly Ni-based austenitic grade materials like Alloy 617, Inconel 625, 740H. High tensile and creep strength, together with other desirable properties at high temperatures such as oxidation and corrosion strength, with relatively low-cost suits P92 material for the application of the components such as main steam pipes, valves and hot reheater pipes, boiler outlet headers, and superheaters pipes. For the application temperature above 620 °C nickel-based alloys are introduced having excellent hot strength, high resistance to creep, good antioxidant property, and corrosion resistance. Alloy 617 is being used for components such as boiler headers and heavy-walled pipework. Alloy 617 was developed by solid-solution and precipitation strengthening of elements Cr, Mo, Co and Al, and Ti, respectively^7^. Al, Ti elements being added to form carbide precipitate (M_23_C_6,_ M_6_C and Ti-C/N)) and intermetallic phase Ni_3_-Al/Ti also known as γ-prime^6^. P92 was developed by adding a small amount of various alloying elements 9%Cr, 0.5%Mo and 1.8%W, and in addition of N, V, Co, Nb and Mn to enhance the solid solution and precipitation strengthening. P92 steel is further used after normalizing and tempering to get the desired mechanical and creep properties due to the development of Cr, Mo, W, or Fe-rich M_23_C_6_^8^, and V and Nb-rich MX carbides and nitrides. During long-term service exposure, intermetallic phases (Z-phase, Laves phase) are also being developed in P92 steel that deteriorates their creep properties^9^.

Different components of the power plants face different steam pressure and temperature. The components that face low operating conditions can be manufactured with low-cost ferritic material rather than expensive austenitic Inconel alloy. Thus, dissimilar metal welds are usually adopted in AUSC power plants depending upon desirable physical, chemical, thermal and mechanical properties of materials, and operating fluid and environment requirements. The dissimilar material successfully joined through many available welding processes but gas tungsten arc welding (GTAW) was adopted most widely because of good weldability, and sound quality in both single as well as multi-pass welding. For the joining of Alloy 617 and P92 steel, the first and most critical factor is to select the suitable filler metal wire. The filler metal should have appropriate mechanical and chemical properties. Mostly nickel-based filler is used for sound welding of ferritic-austenitic dissimilar joints because of better mechanical, chemical, and metallurgical properties in contrast to other filler metals^10,11^. ErNiCrMoCo-1 is used as a filler metal to make Alloy 617 and 9Cr steel joints because of its chemical composition and mechanical properties similar to Alloy 617^7,12,13^. Hosseini et al.^14^ used various fillers (Alloy 617, Inconel 82, 310SS) to make the joint of Alloy 617 and 310SS plates of 12 mm thickness. Mechanical and metallographic tests approved that Alloy 617 filler was the best of the three. In another work, Hosseini et al.^15^ performed the weldability test of Alloy 617 and 310SS dissimilar weldments produced using the Inconel 617, Inconel 82, and 310SS filler. The Inconel 617 filler showed the lowest sensitivity to hot cracking however due to the high induced strain for 310SS filler, it may offer hot cracking in HAZ of 310SS.

The other major issues related to the dissimilar weld metal joint are inequalities in the thermal expansion coefficient (TEC), migration of carbon near an interface, soft delta ferrite formation in 9Cr steel, formation of the filler deficient zone, and Type-IV failure during the service period. The differences in TEC of two different grade materials may lead to the sudden failure of the welded joint caused by cyclic thermal stress generation in metals due to the start and shutdown of the plant^16^. During service, time creep failure at the weld interface might be the problem in the welded joint of steel and Inconel alloy^17^. Some researchers noticed that interfacial failure occurs due to TEC discrepancy and variation of creep strength across the weld interface^18–20^. Carbon-denuded soft-zone formation near the interface might be one of the reasons for interfacial failures of the weld. The carbon migration occurs between the high chromium weld metal and low chromium BM. The carbon shift from low chromium BM to high chromium weld metal. Many authors suggested that Inconel filler would be the better choice to avoid carbon migration as it slows down the diffusion rate of carbon in the weld^10,21^. In addition, overlayers of metal at the interface, having high elements to form carbides, also remedy to avoid carbon-denuded soft zone near the interface^22^. Formation of ferrite in the weldment may also reduce the mechanical properties of weldments. During the weld cooling cycle, the transformation of liquid metal to the solid state under non-equilibrium transformation under high cooling rates promotes ferrite formation close to the interface of the weld or in the HAZ of 9%Cr–Mo steel^23^. The presence of a high weight percentage of W, V and Nb elements (ferrite-former) promotes the formation of stable ferrite in HAZ of 9%Cr–Mo steel^24^. During long-time exposure, the dissimilar weld components mainly fail in Type IV creep conditions. Type-IV fracture is associated with low-stress high temperature creep crack^25^ and mainly initiates in the soft HAZ region of 9%Cr–Mo steel. Parker et al.^26^ proposed that at some locations of HAZ where martensitic transformation not occur generated local high residual stress which adversely affects the creep life and might be one of the reasons for Type IV fracture easily growing in 9%Cr–Mo steel. Some authors found that PWHT has no effect on Type IV failure rather than a bit change in the location of the crack and stated that the heterogeneity in microstructure throughout the weldment plays the main role the Type IV creep cracking^27,28^. The Inconel filler used in dissimilar joining of Inconel alloy and steel enhances the mechanical and creep properties of the welded joint but at the same time, their application leads to the heterogeneity in microstructure along weldments and macrosegregation near the interface of the weld metal and steel. The filler deficient zone (FDZ) appeared adjacent to the fusion line of Inconel filler weld and steel BM in dissimilar weld joints is related to the improper mechanical mixing of filler and BM over the varying melting temperature^29–31^. The FDZ formed in shapes of peninsula, beach, and island at the interface^29^. Compositional similarity of the Inconel alloy with Inconel filler resulted in negligible macrosegragtion at interface. The other problems of such type of the dissimilar welded joint is generation of the high amount of residual stresses. The variation in TEC of both materials play an improntat role in residual stresses evolution. The magnitude of the residual stresses can be reduced by optimizing the weld groove geometry. Investigators emphasize the metallurgical properties, mechanical properties and residual stress variation with the change of angle, size and shapes of grooves for quality weld joints.

Devakumaran et al.^32^ investigated the groove size variation by changing the groove angle for a 25 mm thick plate and found that the elemental composition in the weld zone varies with the variation of groove angle which might be helpful to obtain optimum weld qualities. Variations in the shape of the groove may also affect the width of HAZ of P92 steel^33^. Shuo et al.^34^ optimized the V groove angles of the butt weld joint of a 12 mm thick dissimilar welded plate of P92 steel/super 304H using TP304H as filler and observed 20° groove angle was the optimal groove angle for the weld plate joint, considering mechanical properties for as-welded and PWHT condition. Pratikno et al.^35^ compared butt weld joints of different groove geometry and found better tensile properties for DVG joints than single V groove joints. Li et al.^36^ analyzed the residual stress on the top and the bottom surface of two butt weld joints (single-V groove and double-V groove) made between 10 mm plates of P92 and SUS304 steel, using the hole-drill strain gauge method. Less value of peak tensile residual stress was measured in the DVG joint than single V groove joint design. Mousavi et al.^37^ inspected the residual stress in the U groove and V groove butt weld joint of a 10 mm plate by X-ray diffraction technique, and a low value of peak residual stress was observed for the U-groove geometry. Giri et al.^38^ applied the blind hole technique to measure the developed axial and hoop residual stress in the conventional VG and NVG welded joint of a 25 mm thick circular tube of SS 304LN. High residual stress was observed at the outer surface of the VG joint as compared to NVG welded joint.

From the literature work, it has been summarized that welded joint of Alloy 617 (Inconel 617 alloy) and P92 steel is an integral part of AUSC power units however making a quality weld of these two metals is a challenging task as mechanical behaviour, chemistry and thermos-physical properties of each are different. Limited work has been published on the detailed investigation of Alloy 617 and P92 welded joints. The objective of the work is to study the structural integrity of the Alloy 617 and P92 welded joint, produced using narrow V groove (NVG) and double V groove (DVG) geometry by GTAW process with ERNiCrCoMo-1 filler.

## Experimental details

### Materials and welding details

**Figure 1:**
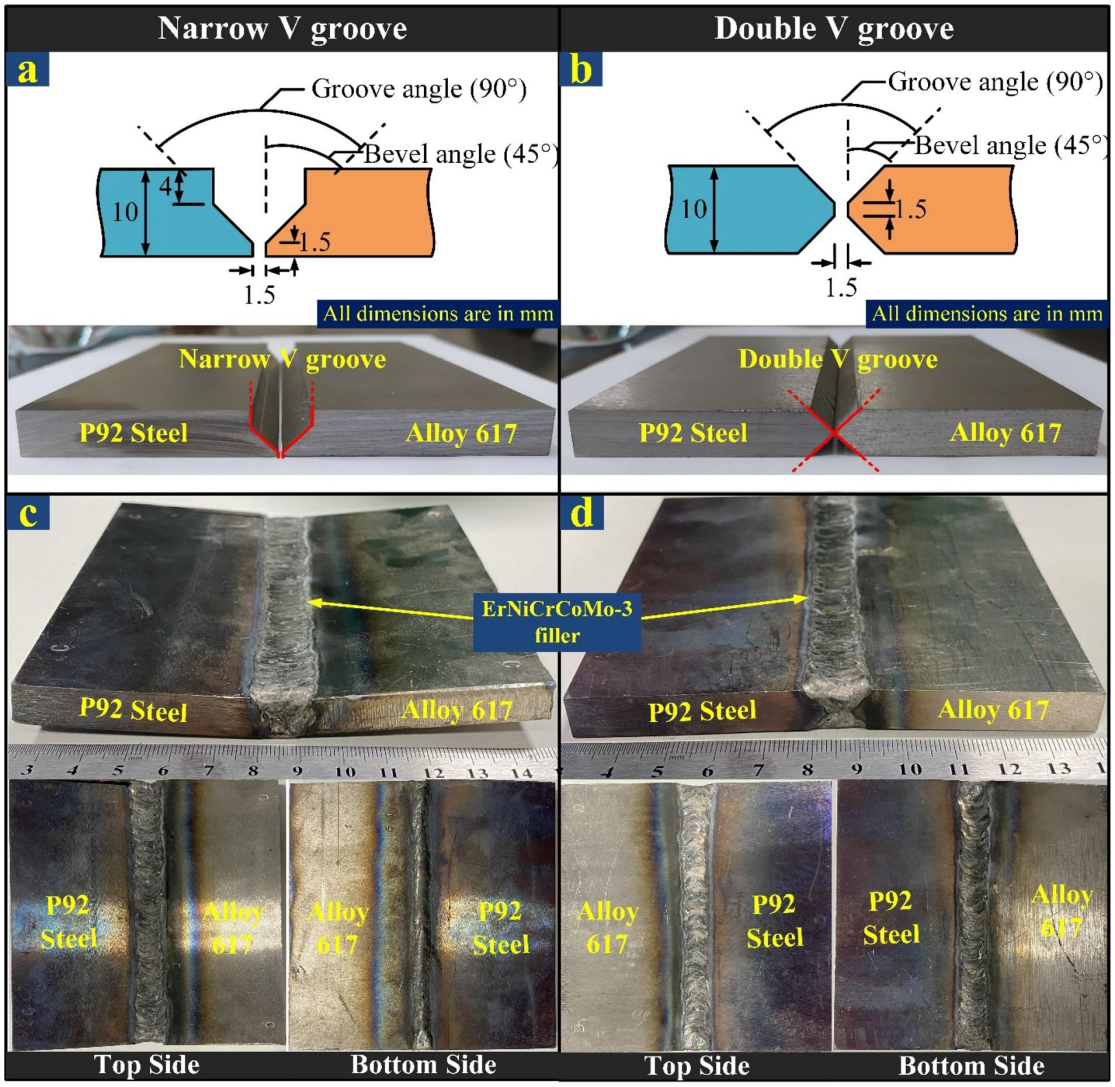
Schematic of groove geometry and machined plate before weld for (**a**) NVG and (**b**) DVG design, (**c**–**d**) welded joint along with top and bottom view of NVG and DVG design, respectively.

The two base metals Alloy 617 (0.058%C, 0.001%Si, 0.013%Mn, 22.30%Cr, 9.11%Mo, 0.33%Ti, 0.016%Nb, 11.40%Co, 1.46%Al, 2.11%Fe, balance Ni; Cr_eq_ = 32.08, Ni_eq_ = 54.70) and P92 steel (0.091% C, 0.23%Si, 0.45%Mn, 8.45%Cr, 0.44%Mo, 1.92%W, 0.001%Ti, 0.32%Ni, 0.042%Nb, 0.18%V, 0.002%B, balance Fe; Cr_eq_ = 9.26, Ni_eq_ = 5.08) plate of dimension 150 mm × 60 mm × 10 mm, were selected for experiment^31^. The edges of both the plates were machined to provide two different designs of groove geometry, namely narrow V groove (NVG) and double V groove (DVG), as shown in Fig. 1a,b. The multi-pass welding was performed by manual tungsten inert gas (TIG) welding process with ERNiCrCoMo-1 filler wire (Max 0.1%C, 1%Si, 1%Mn, 22%Cr, 9%Mo, 0.35%Ti, 1%Nb, 12%Co, 1%Al, 3%Fe, balance Ni; Cr_eq_ = 33.70, Ni_eq_ = 53.05) of 1.6 mm diameter in the surroundings of pure argon (99.99%)^30^. The flow rate of argon was 15 l/min and 10 l/min in shielding and purging conditions, respectively. The complete welding was done with five passes for the NVG and four passes for the DVG butt joints. The welding parameters including voltage, current, and travel speed along with the number of passes along with heat input per unit length are listed in Table 1 for both types of groove designs. Before the start of welding, the root gap was kept at 1.5 mm. The welded plates of NVG and DVG joints, along with their top and bottom view, are displayed in Fig. 1c,d.

**Table 1:** TIG welding parameters.

Welding passes	Welding parameters			Heat input (kJ/mm)	Total heat input (kJ/mm)
	Welding current (A)	Arc voltage (V)	Welding speed (mm/min)		
Narrow V groove					
1. Root	160	~ 16.7	~ 98	0.982	4.638
2. Filling	150	~ 16.2	~ 95	0.921	
3. Filling	150	~ 16.2	~ 95	0.921	
4. Capping	150	~ 16.2	~ 95	0.921	
5. Backing	150	~ 16.2	~ 98	0.893	
Double V groove					
1. Root	160	~ 16.7	~ 85	1.132	4.349
2. Capping	150	~ 16.2	~ 80	1.094	
3. Filling	150	~ 16.2	~ 85	1.029	
4. Capping	150	~ 16.2	~ 80	1.094	

### Mechanical testing, metallurgical characterization and residual stresses measurement

**Figure 2:**
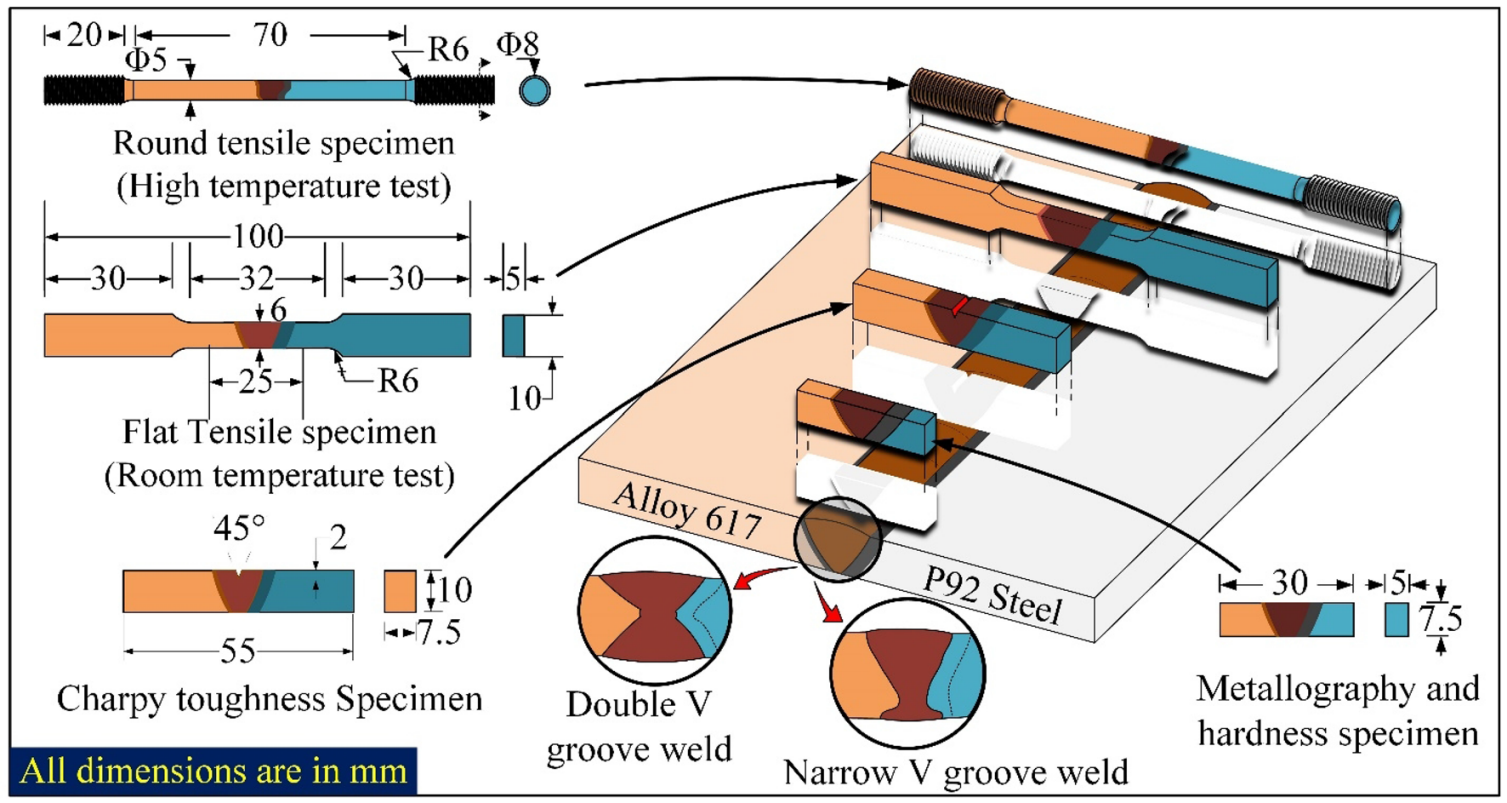
Schematic of the welded plate and extracted samples with dimensions.

**Figure 3:**
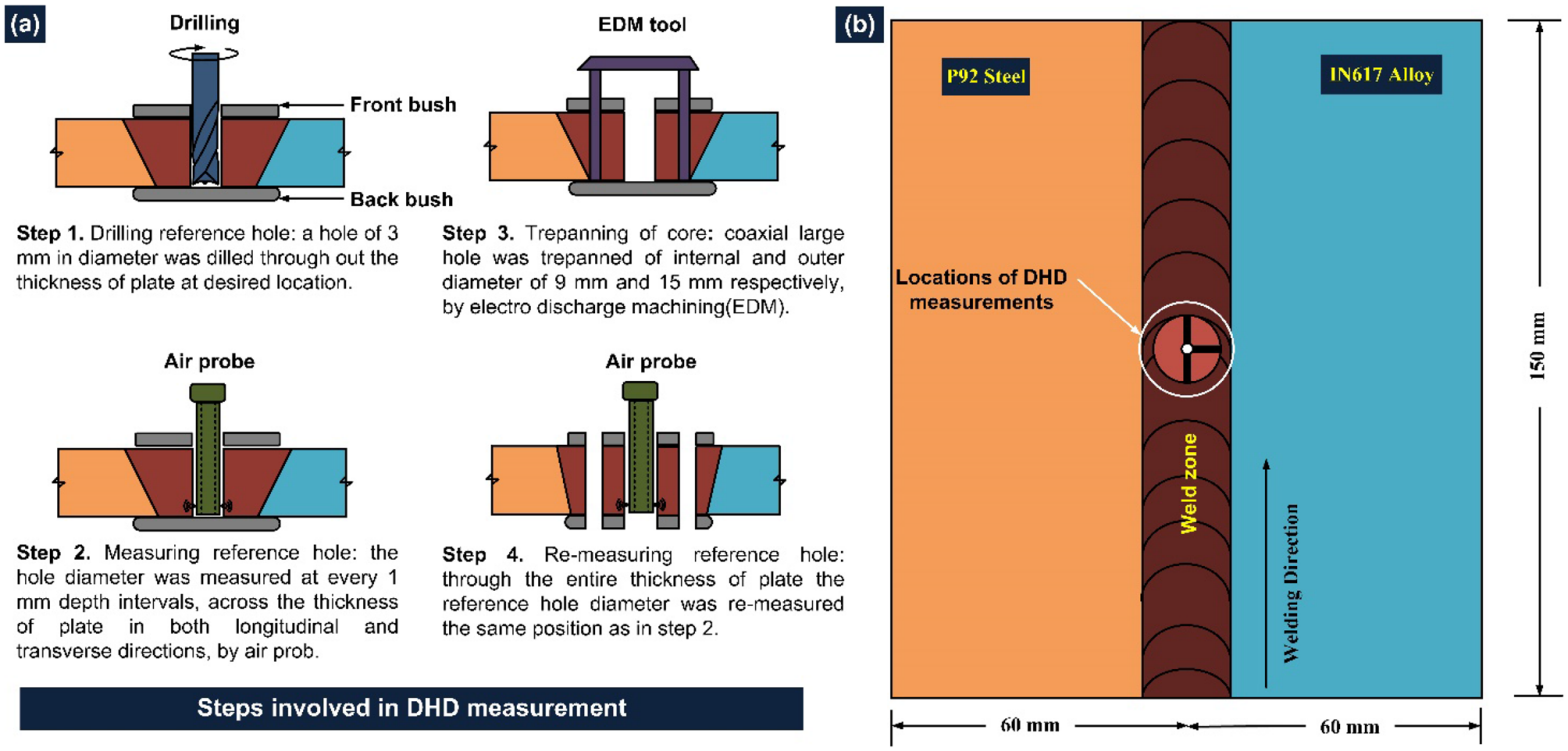
(**a**) Procedure used for residual stresses measurement in DHD technique, (**b**) location for residual stress measurement^41^.

For metallography, specimens of dimension 30 mm × 7.5 mm × 5 mm were machined from both the welded plate which consists of the HAZ region of both P92 and Alloy 617, and weld metal. Metallographic sample preparation was started with the flattening of the sample by using the surface grinder and further finishing was done by using a grinding and polishing machine with silicon carbide papers grit size from 240 to 2000, followed by cloth polishing with fine alumina powder to provide mirror finish. P92 base and HAZ were etched with Vilella’s reagent. Weld metal, Alloy 617 base and HAZ were etched with electrolytic oxalic etching solution (10 g oxalic acid, 100 ml H_2_O). For grain size distribution, an optical microscope was used. Further detailed investigation of the weld interface and elemental distribution in the weld metal and HAZ, field emission scanning electron microscope (FESEM) was used. To characterize the fusion interface and weld metal for elemental diffusion and chemistry of the phases, FESEM is equipped with EDS and electron probe microanalyzer (EPMA). Room temperature (RT) tensile test, high temperature (HT) tensile test, Charpy toughness (CT) test, Vickers hardness (VH) test, and Residual stress measurement were considered under mechanical testing for this study. For RT tensile test, three sets of the flat sub-size dog bone sample of 100 mm length were extracted as per ASTM E8/E8M- 16a standard (Fig. 2). To evaluate the tensile properties of the welded joint, a transverse specimen is also machined by Kulkarni et al.^39^ and Mittal and Sindhu^40^. For HT tensile test, a round specimen (as per ASTM standard E8/E8M-13a) was produced with a length of 110 mm and a gauge diameter of 6 mm (Fig. 2). RT and HT tensile testing was performed at 25 °C and 650 °C with an extension speed of 1 mm/min in a Shimadzu AG–X tensile tester of capacity 100 kN. Specimen of size 55 mm × 10 mm × 7.5 mm was used for the CT test with the V notch at the top of the weld metal (Fig. 2). The VH indent was taken along the weldment at the load of 0.5 kg with a dwell time of 10 s. Moreover, residual stress was estimated with the deep hole drilling (DHD) technique, along the thickness of the plate. The procedure involved in the DHD techniques is mentioned in Fig. 3.

## Results and discussion

### As-received material

**Figure 4:**
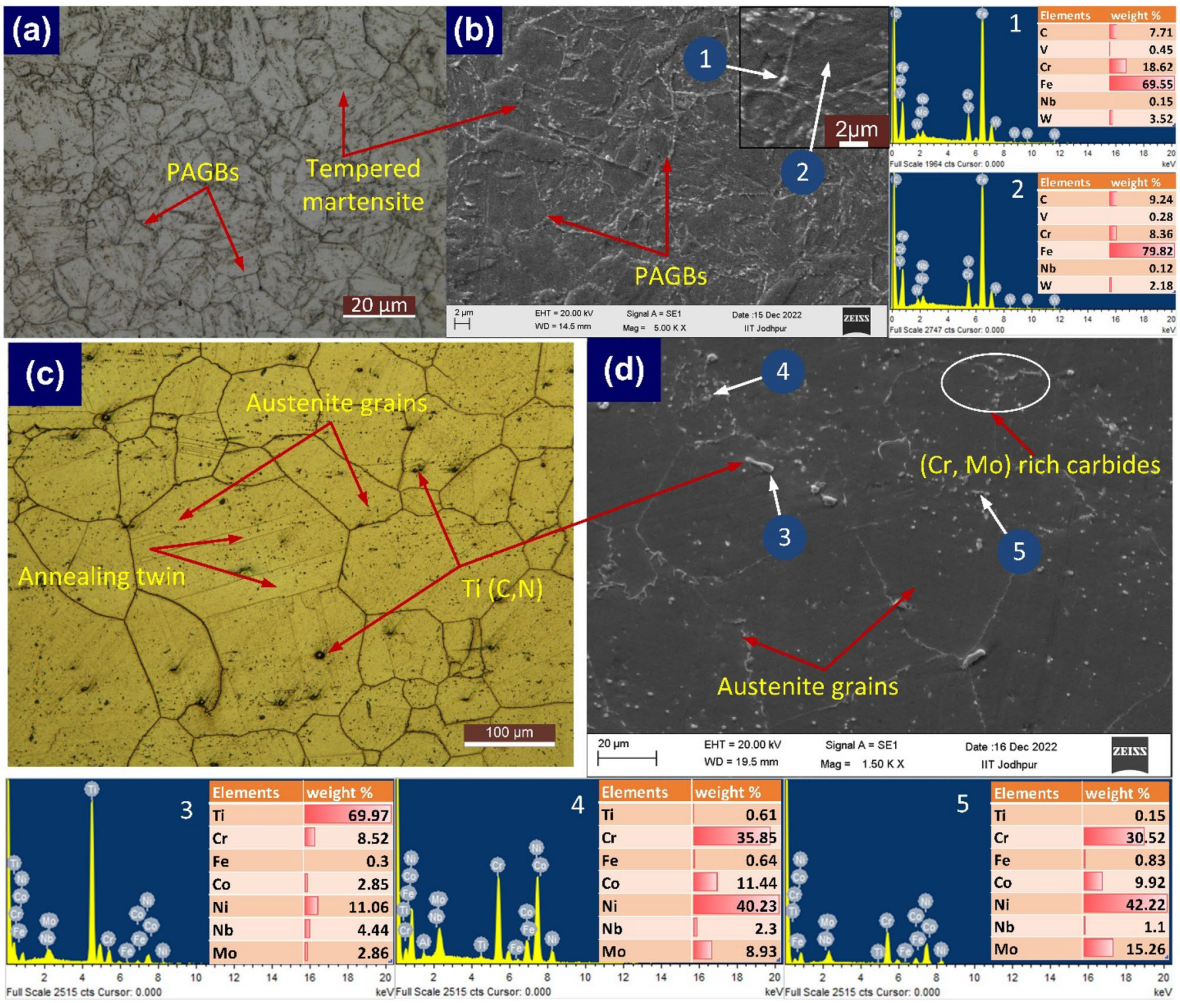
Base metal microstructure: P92 (**a**: optical, **b**: SEM); Alloy 617 (**c**: optical, **d**: SEM) and EDS spectra.

The BM optical microscopic (OM) and scanning electron microscopic (SEM) microstructure of Alloy 617 and P92 steel are shown in Fig. 4. The tempered martensitic microstructure of P92 steel consists of prior austenite grains (PAGs), and packets of lath blocks within PAGs (Fig. 4a), and precipitate along boundaries and within the matrix (Fig. 4b). The PAGs was 12 ± 8 µm. The precipitates were rich in Cr and W as analysed by EDS (arrow 1 in Fig. 4b) and it could be the phase of M_23_C_6_ enriched with Cr and W^42^. The austenitic microstructure of BM of Alloy 617 consists of austenitic grain, twins, and randomly distributed precipitates (Fig. 4c). The maximum, minimum and average austenite grain sizes were 151 µm, 61 µm and 100 ± 30 µm^31^. The block shape of coarse titanium carbide precipitates was observed to be distributed heterogeneously in the alloy. The random distribution of the fine precipitates along boundaries and inside the matrix is found from the SEM image (Fig. 4d). The block shape particles were Ti–rich carbides (69.97 wt.% Ti) (arrow 3) while fine precipitates were Mo and Cr-rich M_23_C_6_ (35. wt% Cr and 8.93 wt% Mo) (arrow 4) carbides, and Mo rich M_6_C (15.26 wt% Mo) (arrow 5) precipitates^31^.

### Weldments characterization

**Figure 5:**
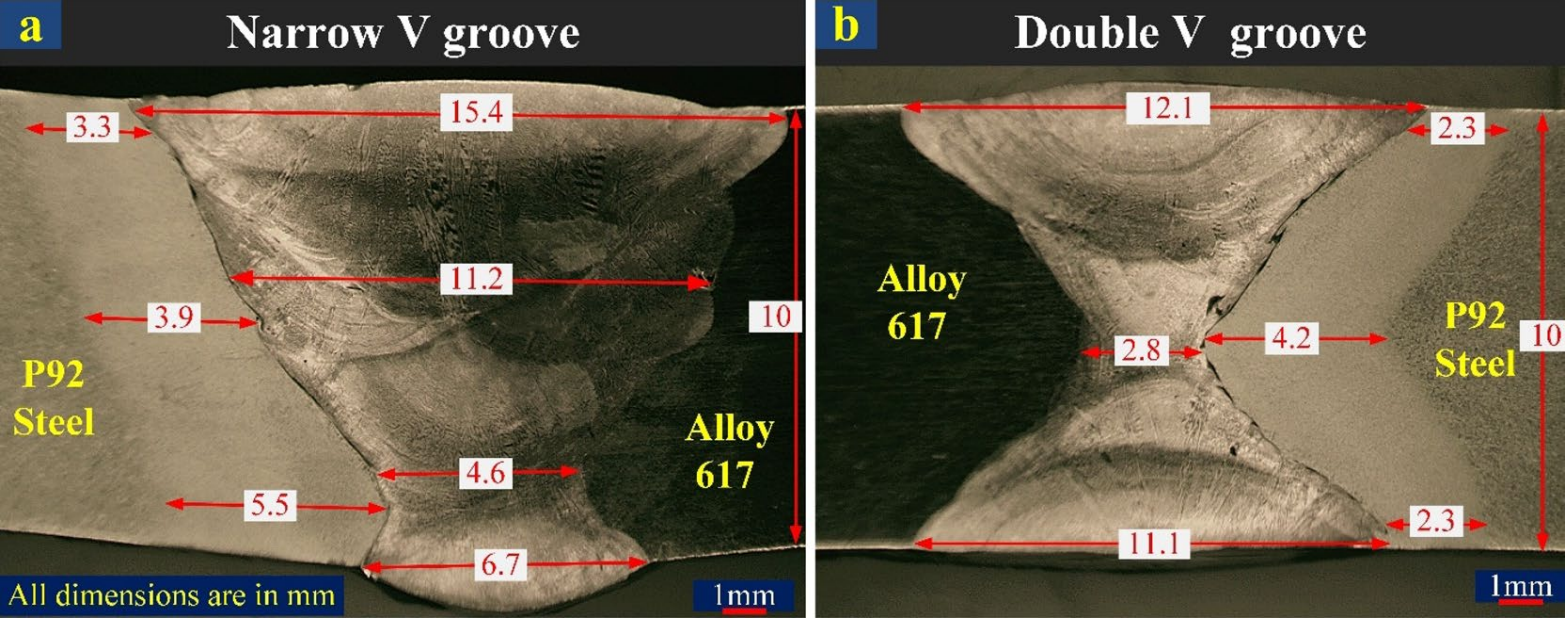
Macrograph of (**a**) NVG and (**b**) DVG butt joints.

**Figure 6:**
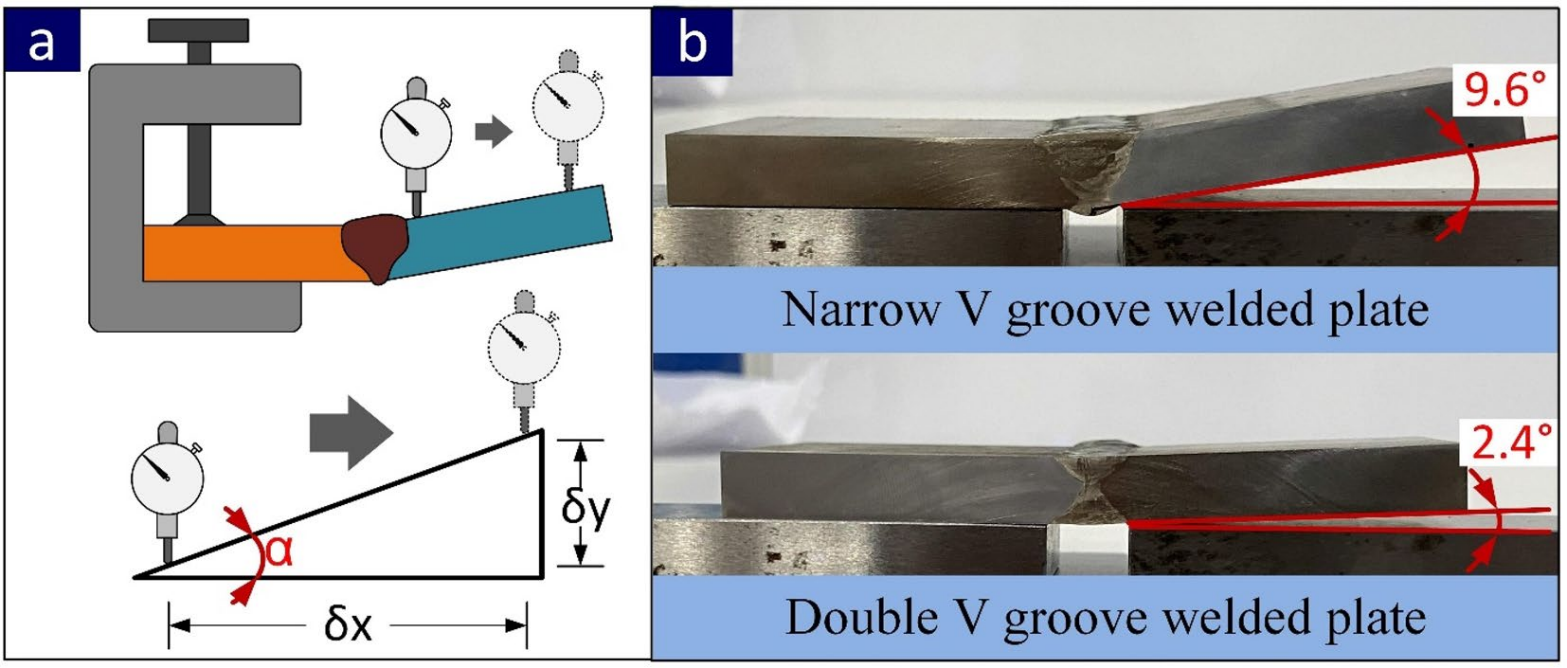
(**a**) Schematic of angular distortion measurement setup (**b**) distorted welded plate of NVG and DVG joint.

The visual inspection and radiography testing of the welded plate was performed before metallographic characterization and mechanical testing to inspect the soundness of the weld. The good quality weld free from defects and cracks is inferred from test results. The weld bead was also uniform with the negligible presence of undercut, underfill, pits, cracks and pores. The macrograph of both joints is displayed in Fig. 5a–b with a clear view of the weld zone and HAZ. The root, filling and capping passes are also visible in the micrograph. The post-weld groove opening and root gap were 15.4 mm and 4.6 mm, respectively for NVG, and a groove opening of 11.6 mm (average) and root gap of 2.8 mm was measured for the DVG joint. The width of HAZ varies from top to bottom and it was 2.3 mm at the top and bottom and 4.2 mm at the weld centre for DVG. In NVG, HAZ width was 3.3 mm and 5.5 mm at the top and bottom and 3.9 mm in the weld centre. The plate was in unconstrained condition to observe the effect of the weld groove geometry on angular distortion (α). The residual stress measurement was also performed in the unstrained condition of the welded plate. The angular distortion measurement was performed as per the schematic given in Fig. 6a. The average angular distortion in the NVG joint was 9.6° and get reduced to 2.4° in the DVG joint as mentioned in Fig. 6b. The Cr_eq_ and Ni_eq_ values of 33.70 and 53.05 were calculated for ERNiCrCoMo-1 filler. For weld metal, Cr_eq_ and Ni_eq_ values were 31.57 and 53.26, respectively. An optical emission spectrometer (Metavision Make, model 1008i) was employed to examine the chemical composition of the weld metal. The analysis was performed at the centre of the weld metal. The composition was observed as follows: 0.06%C, 0.079%Si, 0.012%Mn, 21.49%Cr, 9.21%Mo, 0.37%Ti, 51.45%Ni, 0.015%Nb, 10.20%Co, 2.25%Al, and 4.54%Fe. The Cr_eq_ and Ni_eq_ values for filler metal and weld metal affirmed that weld metal is solidified in austenitic mode (L → L + ϒ → ϒ)^43^.

#### XRD analysis

**Figure 7:**
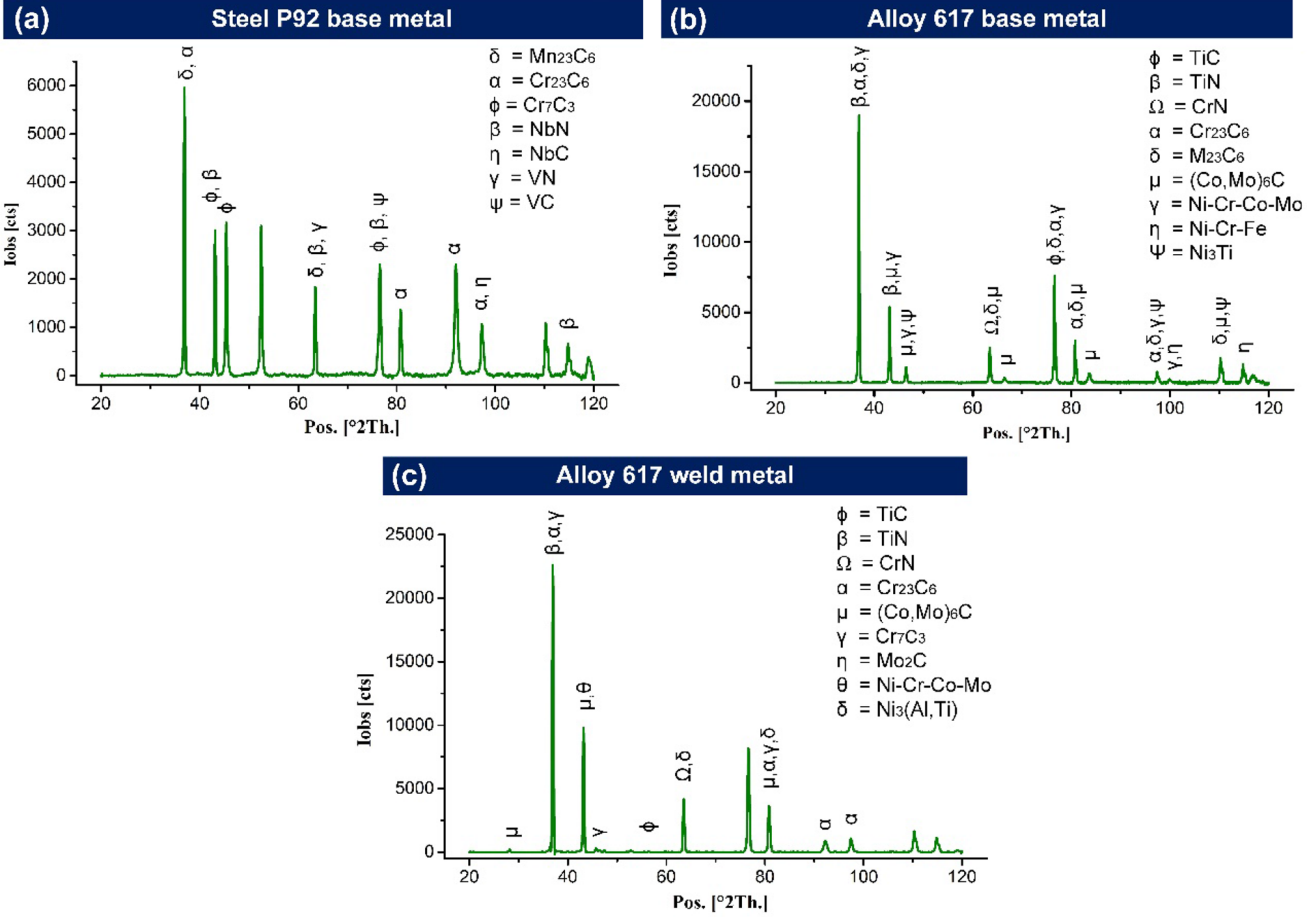
X-ray diffraction diagram for (**a**) P92 BM (**b**) Alloy 617 BM (**c**) weld metal.

The EDS spectrum was taken to confirm the phase and their composition in BMs and weld metal. The phases detected using the EDS spectrum were further confirmed using XRD analysis. In P92 BM, major peak of Cr_23_C_6_, Mn_23_C_6_, Cr_7_C_3_, Nb(C, N), and V(C, N) was observed (Fig. 7a). The peak of M_23_C_6_, Ti(C, N), CrN, (Co, Mo)_6_C, ϒ’ (Ni_3_Ti), ϒ(Ni–Cr–Co–Mo) and ϒ(Ni–Cr–Fe) was reconized in Alloy 617 BM (Fig. 7b). In ERNiCrCoMo-1 weld, Cr_23_C_6_, CrN, Ti(C, N), (Co, Mo)_6_C, ϒ’ (Ni_3_(Al,Ti)), Cr_7_C_3_, Mo_2_C, ϒ(Ni–Cr–Co–Mo) and ϒ(Ni–C–Fe) was reconized at differet peaks as mentioned in Fig. 7(c). ϒ(Ni–Cr–Co–Mo) and ϒ(Ni–Cr–Fe) are the peaks that corresponded to the austenitic matrix of Alloy 617 BM and weld metal. The different carbide phases in BMs and weld metal strengthen the grain boundaries by pinning their movement and increasing the creep strength. In P92 steel, Cr rich M_23_C_6_ phase increases the creep strength by clogging the grain boundary movement while precipitates of V and Nb (VC, NbC, VN, and NbN) clog the dislocation movements and enhances the creep strength. The precipitates enrich with V and Nb also show higher thermal stability at a temperature above 650 °C than the M_23_C_6_. The TiC and TiN phases in Alloy 617 BM and weld metal reduces the coarsening of the grain boundaries by pinning their movement under high-temperature service conditions. The ϒ’ phase (Ni_3_(Al,Ti)) is mainly observed in Ni-based alloys and provides precipitation strengthening to the matrix. They offer higher stability even at a temperature higher than 700 °C. The formation of the ϒ’ phase in the weld metal is predominantly attributed to the slow cooling rate during welding. Conversely, the rapid cooling causes the dissolution of the ϒ’ phase in the austenitic matrix. The phases were rich in Cr and Mo (M_23_C_6_ and M_6_C) provide strengthening to the inter-dendritic boundaries of the weld metal. The size and distribution of these phases are analysed using the FESEM image. The size, composition and distribution of phases along boundaries and within the matrix play a vigorous role in deciding the mechanical properties and creep strength of the BM and weld metal. Saini et al.^44^ and Sun et al.^45^ also confirmed the existence of similar phases in P92 and Alloy 617 BMs and weld metal.

#### Weld interface characterization

**Figure 8:**
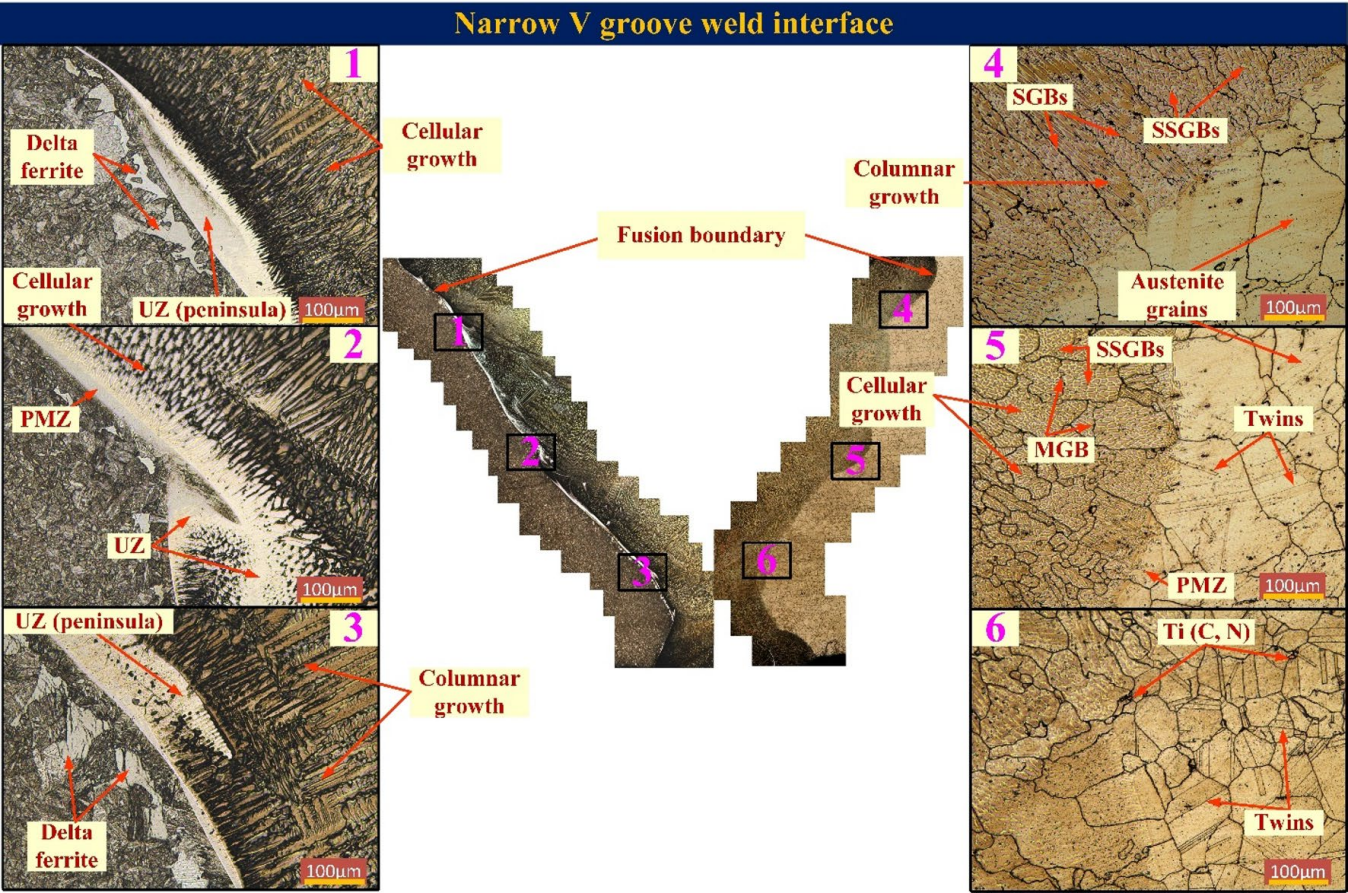
Optical image microstructures of 10 mm area in Fig. 5, high magnification image of different regions form along fusion boundary on either side for NVG joint.

**Figure 9:**
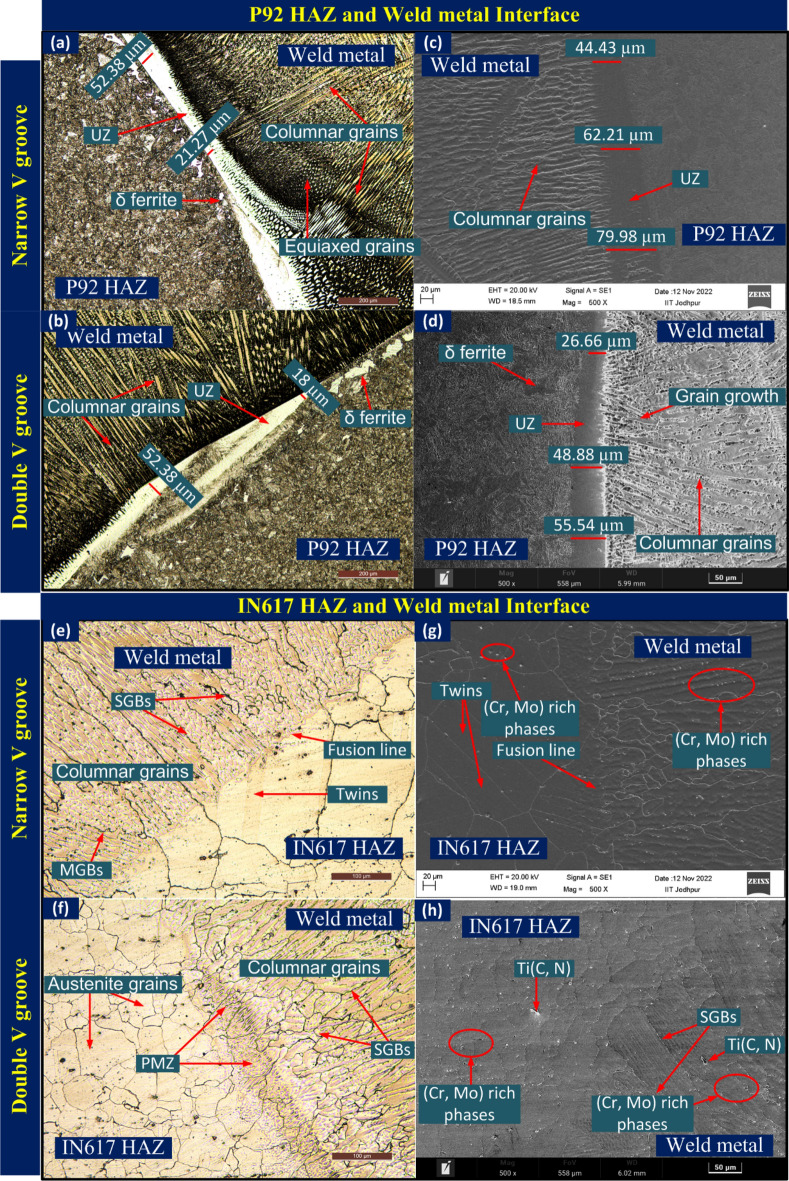
Interface between P92 steel and ERNiCrCoMo-1 weld metal characterizing using optical and SEM image showing extensive UZ: (**a**, **c**) NVG joint, (**b**, **d**) DVG joint; interface between Alloy 617 and ERNiCrCoMo-1 weld metal with no detectable UZ; (**e**, **g**) NVG joint, (**f**, **h**) DVG joint.

The macrosegregation near the interface is one of the major problems as studied in earlier works. The interface comprised of the weld metal, HAZ and unmixed zone. Figure 8 shows the microscopic metallographic microstructure of the welded joint along the fusion boundary in a 10 mm area (along plate thickness) for both sides of the weld metal for NVG joints along with some enlarged high magnification images. The image was captured at different locations corresponding to welding passes. The microstructure near the fusion line has been described using various terminologies like filler deficient zone/beach/peninsula/island^46^, unmixed zone (UZ)^46–48^, featureless zone^49^, transaction zone^10^ and weld metal swirl^50^. In the present investigation, the terminology (beach, i.e. UZ, peninsula and island) proposed by Kou and Yang^51^ was used to define the feature adjacent to the fusion line, which was attributed to severe macrosegregation due to differences in the composition of the filler and BM. The macrosegregation has reported a negative impact on the performance of the welded joint such as solidification cracking^52^, poor corrosion resistance, loss of ductility^53^, hydrogen cracking^54^ and reduction in creep strength. The weld metal micrograph near the fusion boundary, macrosegregation, and micrograph of the HAZ depends on many parameters like cooling rate, filler metal composition, and the number of welding passes. The interface microstructure includes the beach parallel to the fusion boundary on the P92 steel side (Fig. 8), the peninsula connected to the fusion boundary (region 1–3 in Fig. 8), the island within the weld metal, and the partially melted zone (PMZ) along Alloy 617 fusion boundary. A distinct region of the beach spread along the fusion boundary was observed on the P92 steel side, as depicted in Fig. 8. The uneven distribution of beach, peninsula and island structure along the fusion boundary was noticed. The minimum beach size was 4 µm corresponding to the capping pass. However maximum was 79.98 µm near to filling pass. The size varies between 18 to 52.38 µm for filling passes. The size is measured in the range of 44.43–79.98 µm for the NVG joint and 22.66–55.44 µm for the DVG joint, as shown in Fig. 9. The beach formation is a result of improper mixing and solidification of the molten P92 BM with non-matching and over alloyed ERNiCrCoMo-1 filler^52^. The convection and scouring of the weld pool cause the entry of the insufficiently mixed P92 BM and ERNiCrCoMo-1 filler into weld metal which results in the formation of a peninsula and island by undercooling^46^. The PMZ is the region formed within the HAZ where BM experienced a temperature below liquidus but above solidus^55^. The other important noticeable thing along the P92 side was the formation of δ ferrite in coarse-grained HAZ (CGHAZ) in varying sizes, shapes, and densities, similar to other observations made by Mittal and Sidhu^40^. The higher density of δ ferrite adjacent to backing and capping passes (Region 1 and 3 in Fig. 8) and poor adjacent to root and filling passes (Region 2 in Fig. 8) are witnessed. The reheating result of capping and backing passes increases the temperature of the HAZ corresponding to root and filling passes which results in the dissolution of the δ ferrite. The δ ferrite has poor stability at a temperature below 1250 °C and complete dissolution occurs at a temperature of about 1050 °C^56^. The δ ferrite dissolution mainly starts at a temperature of about 887 °C, i.e. A_c3_ temperature of P92 steel. In the Fe–Cr matrix of P92 steel, the depletion of Cr promotes the development of the Cr-rich δ ferrite phase. The depletion of the Cr from a particular region of the martensitic CGHAZ matrix of P92 could degrade the oxidation resistance. During the heating effect of subsequent passes, the dissolution of the δ ferrite from the CGHAZ region corresponding to root and filling passes is also governed by the diffusion of the Cr and W from the δ ferrite to the austenitic matrix. As compared to the P92 steel side, the other side of the fusion boundary, i.e., Alloy 617, shows negligible macro segregation (Fig. 8). A region of PMZ and a very narrow region of the beach is inspected on Alloy 617 side for both groove welded joint. The closeness in melting point and composition of the Alloy 617 BM and filler resulted in more dilution and hence no considerable beach formation is detected. The influence of the welding passes on solidification behavior of the weld metal is also studied. The weld metal with columnar and cellular grain structure having precipitates along boundaries are observed in the enlarged view images. The weld metal completely austenitic in nature confirms the austenitic mode of the solidification (A type). Mostly Ni based filler solidifies in A type because of the remarkably amount of Ni content in filler wire. The weld metal characteristic includes the solidification sub-grain boundary (SSGB), the solidification grain boundary (SGB), and the migrated grain boundary (MGB)^57,58^. The boundaries are labelled in the weld metal microstructure adjacent to fusion boundary (Fig. 8). The MGBs were present in weld metal corresponding to filling pass (region 5 in Fig. 8) which are often reasoned as the origin of the ductility-dip cracking^57^. The formation is associated with multi-pass welding cycles which pushes the boundaries to migrate to achieve the stable structure by lowering the energy associated with SGBs.

The enlarged view of the interface is displayed in Fig. 9. Interface between P92 steel and ERNiCrCoMo-1 weld shows an extensive region of the UZ for both NVG (Fig. 9a,c) and DVG (Fig. 9b,d) joint. This could be due to the differences in chemistry and the melting point of filler and BM. However, no detectable UZ formation is witnessed at the interface of Alloy 617 BM and weld for both NVG (Fig. 9e,g) and DVG (Fig. 9f,h) joint. This could be attributed to more dilution and closeness in chemical composition and the melting point of the BM and filler. The SEM image is used to display the segregation in the weld metal and also shows the variation in size of the UZ along the fusion line (FL). As it is known that welding of the dissimilar base, i.e. P92 steel and ERNiCrCoMo-1 weld creates a significant difference between weld metal near FL and bulk weld metal in terms of segregation, i.e. composition, microstructure and mechanical properties. The competitive growth in weld metal adjacent to FL is witnessed from an optical image^59^. At the time of solidification, grains are intended to grow in the direction perpendicular to the pool boundary which is also a direction of maximum heat extraction. This facilitates the easy growth of the grains and crowds out less favourably oriented grains. From the interface, a columnar and cellular type of growth is detected in the weld metal and at Alloy 617 interface grain grows from BM into weld metal (Fig. 9e–h) and is roughly aligned perpendicular to FL. To understand the elemental diffusion and segregation near the FL, an EDS and EPMA study has been performed and the results of same are presented below.

**Figure 10:**
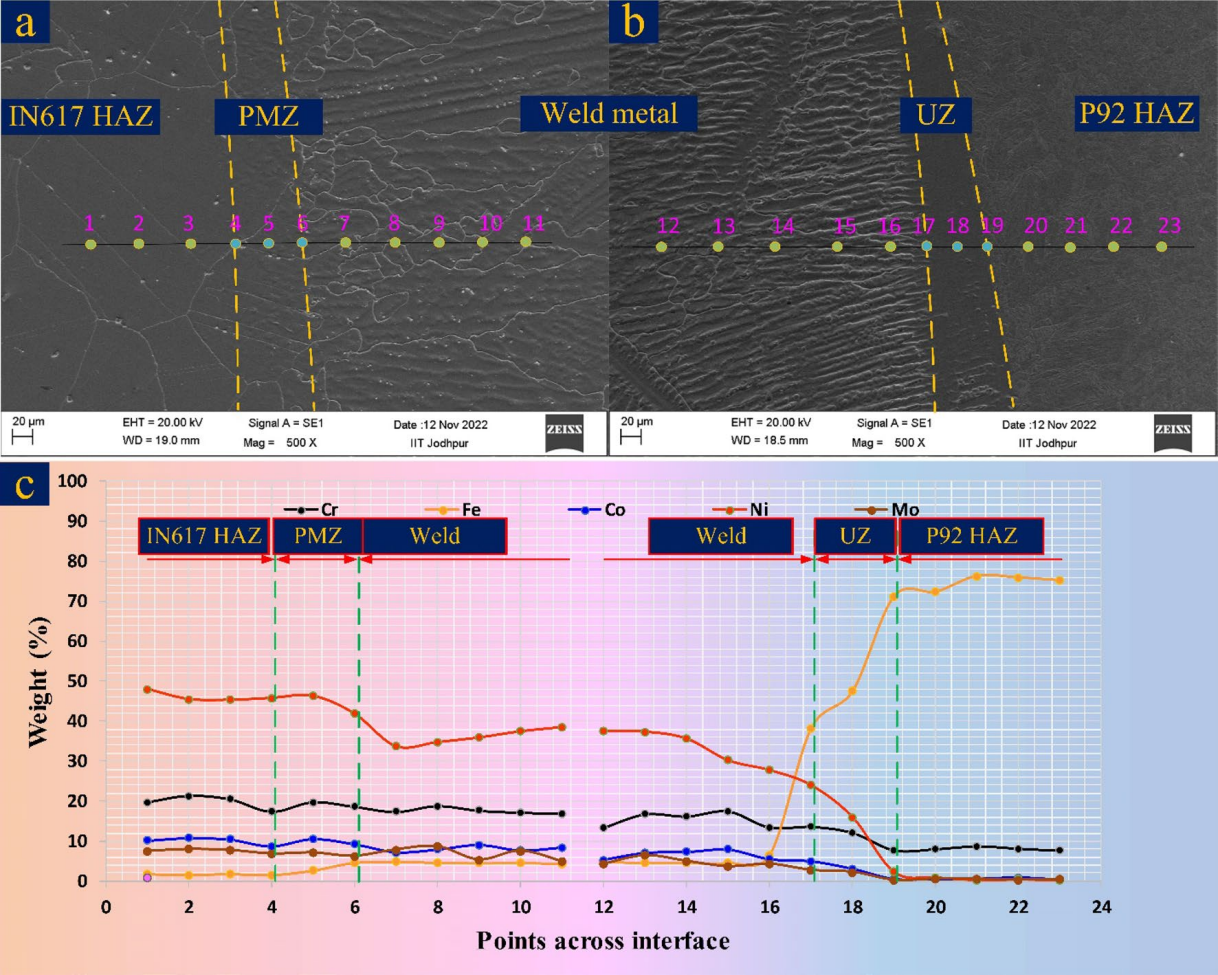
Region of interface selected for EDS mapping (**a**) P92 steel, (**b**) Alloy 617 and (**c**) EDS mapping showing the composition profile across PMZ (Alloy 617 interface) and unmixed zone (P92 steel).

**Figure 11:**
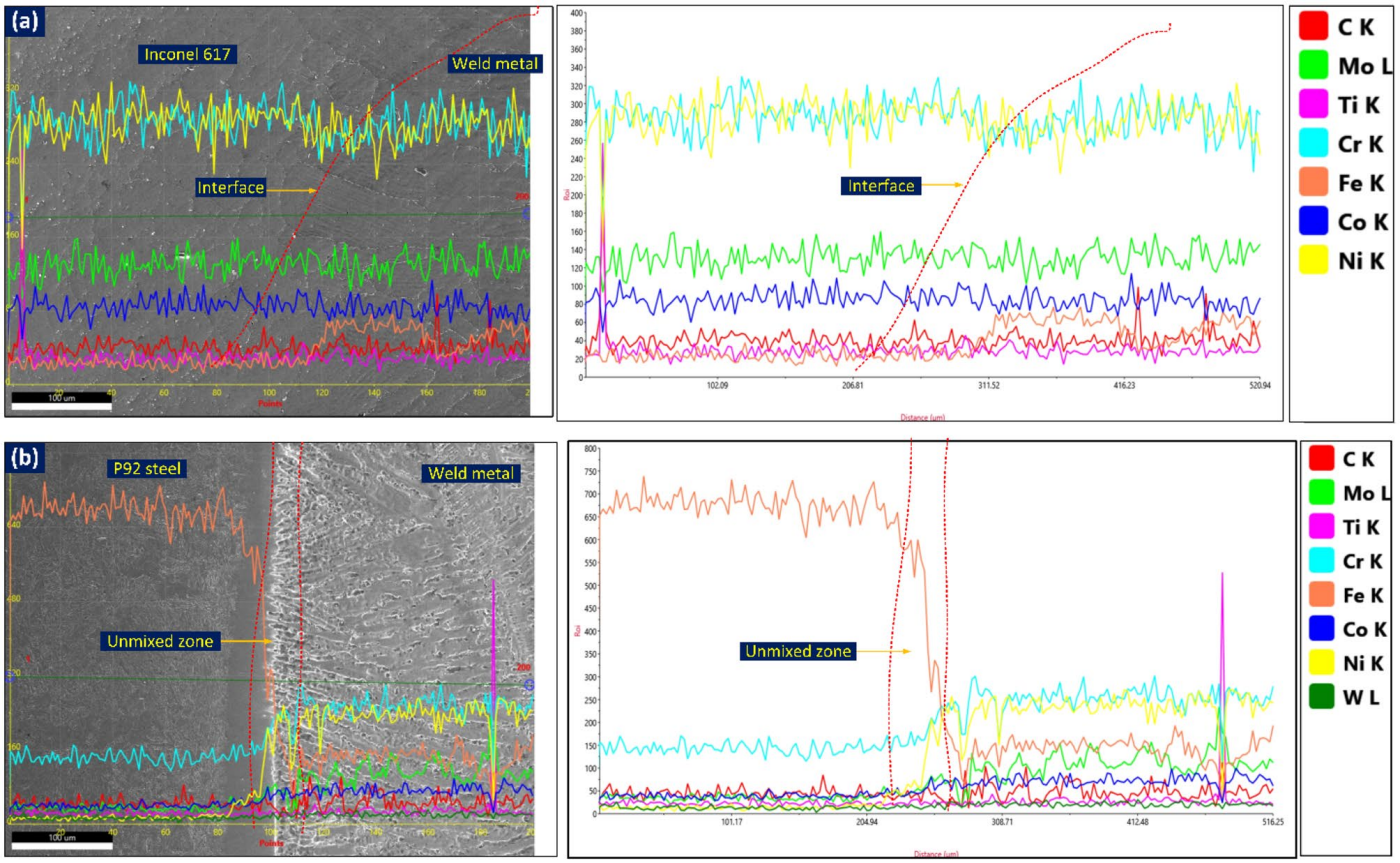
EDS map showing (**a**) negligible elemental variation at the interface of Alloy 617, (**b**) significant variation in the area of UZ.

The mechanical performance of the welded joint largely depends on the characteristic of the interface which includes macrosegregation, element diffusion and segregation. The EDS map of the interface is displayed in Fig. 10a–c which includes Cr, Fe, Co, Ni, and Mo. The map displays the change in element weight percentage corresponding to the movement from Alloy 617 to weld metal to P92 steel. The EDS map approves the major concentration of the Mo, Ni, Co and Cr in PMZ present at the Alloy 617 interface. However, the concentration was between Alloy 617 BM and weld metal. The map shows the major change in concentration of the Fe as moves from the Alloy 617 BM to weld metal. A major variation in element concentration is witnessed near the interface of P92 steel (Fig. 10c). The EDS shows the major variation of Fe, Co, Ni, Cr and Mo concentration between P92 steel and ERNiCrCoMo-1 weld, and the weld shows a higher concentration of Co, Ni, Cr and Mo. However, a major change in concentration is detected for Ni and Fe. Across the interface of P92 steel, the weight % of Ni varies between 0.25% (P92 steel) and 37.48% (weld metal) while Fe varies from 4.58% (weld metal) to 76.25% (P92 steel). The weight % of Fe and Ni in UZ (area between BM and weld metal) is 46.44% and 15.59%, respectively. The composition of the P92 BM and filler indicates the difference in weight percentage of the Fe, Cr, Co, Ni and Mo. So the activity of the migration of these elements across the interface could be expected high as compared to elements like Ti, and W. From Fig. 10c, the migration of the Fe from steel BM to ERNiCrCoMo-1 weld and migration of Ni, Co, Mo and Cr from ERNiCrCoMo-1 weld to steel BM is inferred. The concentration of the Mo, Cr and Co in the UZ is 2.26%, 12.15% and 3.06%, respectively which are relatively higher than the adjacent P92 steel BM. The concentration of Ni in weld metal near the interface (point 7: 33.76%, point 16: 27.78%) is measured lower than the concentration in weld metal (51.45%) observed using an optical emission spectrometer which could be due to the migration of the Ni from weld metal. Similarly, the weight percentage of Co, Mo and Cr in weld metal near to interface is measured lower than the actual weight percentage observed in weld metal using an optical emission spectrometer. This variation in the concentration of the weld metal creates inhomogeneity in the mechanical properties of the weldments. A strong influence of the element diffusion on the end performance of the welded joint has already been confirmed from previously reported work. The Cr diffusion near the FL promotes the formation of the Cr-rich phases which could be the M(Cr, Fe)_23_C_6_ and Cr_7_C_3_ and phase of Ni–Cr–Fe as addressed by Mittal et al.^40^. The elemental line map for the same has also been conducted and displayed in Fig. 11. A smooth elemental profile is witnessed across Alloy 617 interface (Fig. 11a). A minute increase in Fe peak is evident from Fig. 11a which is similar to EDS line map. The uniform peak of the elements at the interface confirms the higher dilution which could be attributed to the use of matching filler metal. Like the EDS line map, the line map of elements also shows the sharp composition gradient of elements Cr, Ni, Co, Mo and Fe (Fig. 11b). In the region of UZ, a drastic drop in the concentration of Fe and drastic increase in the concentration of Cr and Ni is witnessed (Fig. 11b) when moving from P92 to weld metal. The elements Mo and Co also present the increase in concentration from P92 to weld metal. The increase in the intensity of C in the area of weld metal near UZ is also evident across the P92 interface. however, it is difficult to reach any conclusion about C diffusion from that EDS map. The UZ which has higher Fe content, the structure will be BCC, i.e. similar to P92 BM^40^.

**Figure 12:**
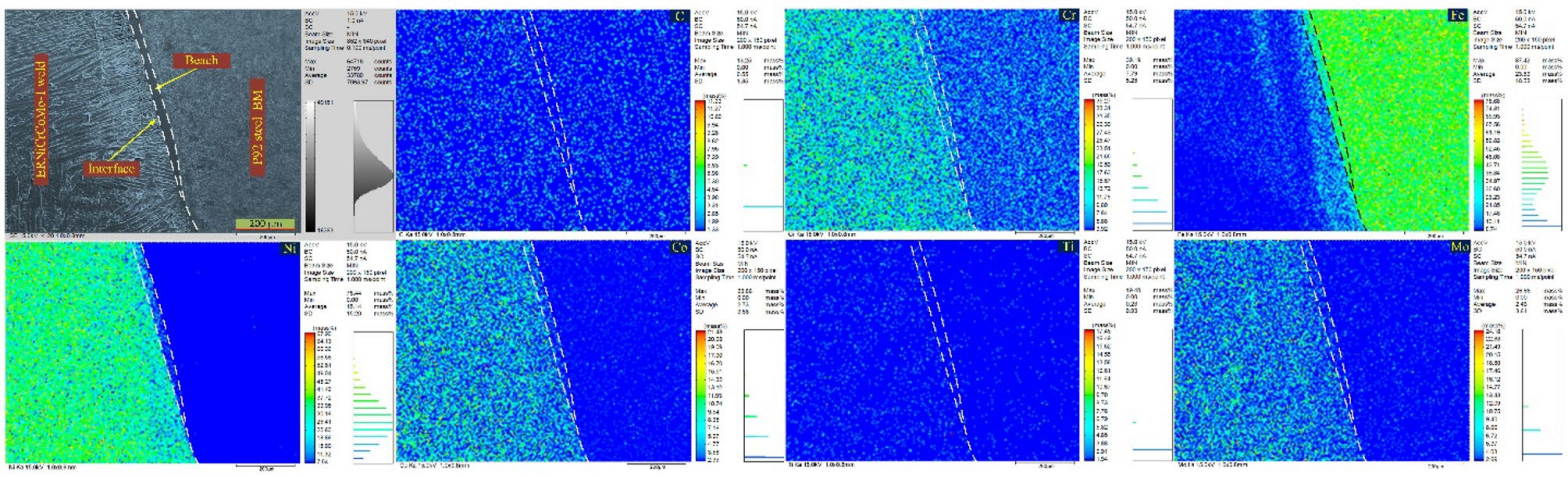
EPMA map confirming diffusion of elements across the interface of P92 steel.

**Figure 13:**
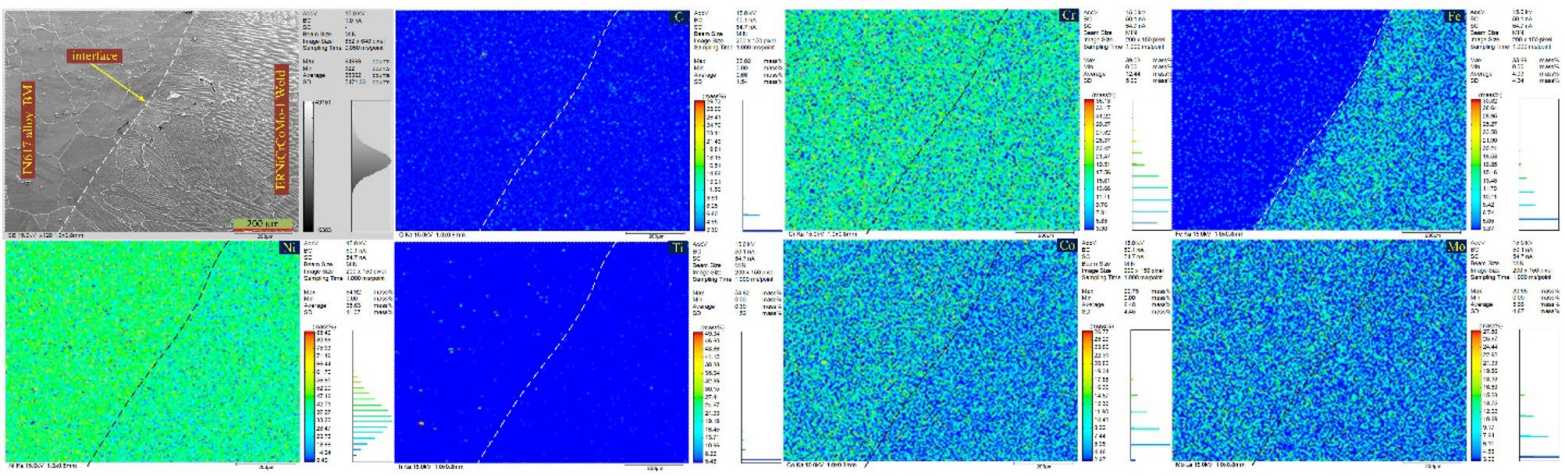
Interface showing minor diffusion at the interface of Alloy 617.

To observe the C diffusion near the interface of P92 steel, an EMPA study was also conducted and the results are presented in Fig. 12. The diffusion of the elements (C, Fe, Cr, Co, Ni and Mo) along FL is witnessed from the EMPA map. The uniform distribution of the C and Cr are confirmed from EMPA on Alloy 617 interface (Fig. 13). The variation in concentration of the Fe, Ni, Co and Mo is witnessed from EMPA and it could be due to the minor difference in composition of Alloy 617 BM and filler metal. The higher concentration of the Ti in Alloy 617 BM certified the availability of the Ti(C, N) phases. From EDS point analysis, line map and EPMA study, diffusion of the Fe, Ni, Cr, Co and Mo at the interface of P92 steel are evident. However change in concentration gradient for Fe and Ni was sharp than the Cr, Co and Mo. The availability of Mo in UZ could be due to their poor solubility in the austenitic matrix. The poor diffusion rate of Mo in the austenitic matrix also ensures their availability in UZ. No segregation tendency for Cr, Fe, Ni, Mo and Co is observed at the interface of Alloy 617 due to the similarity content of the Alloy 617 and weld metal.

#### Characterization of HAZ

**Figure 14:**
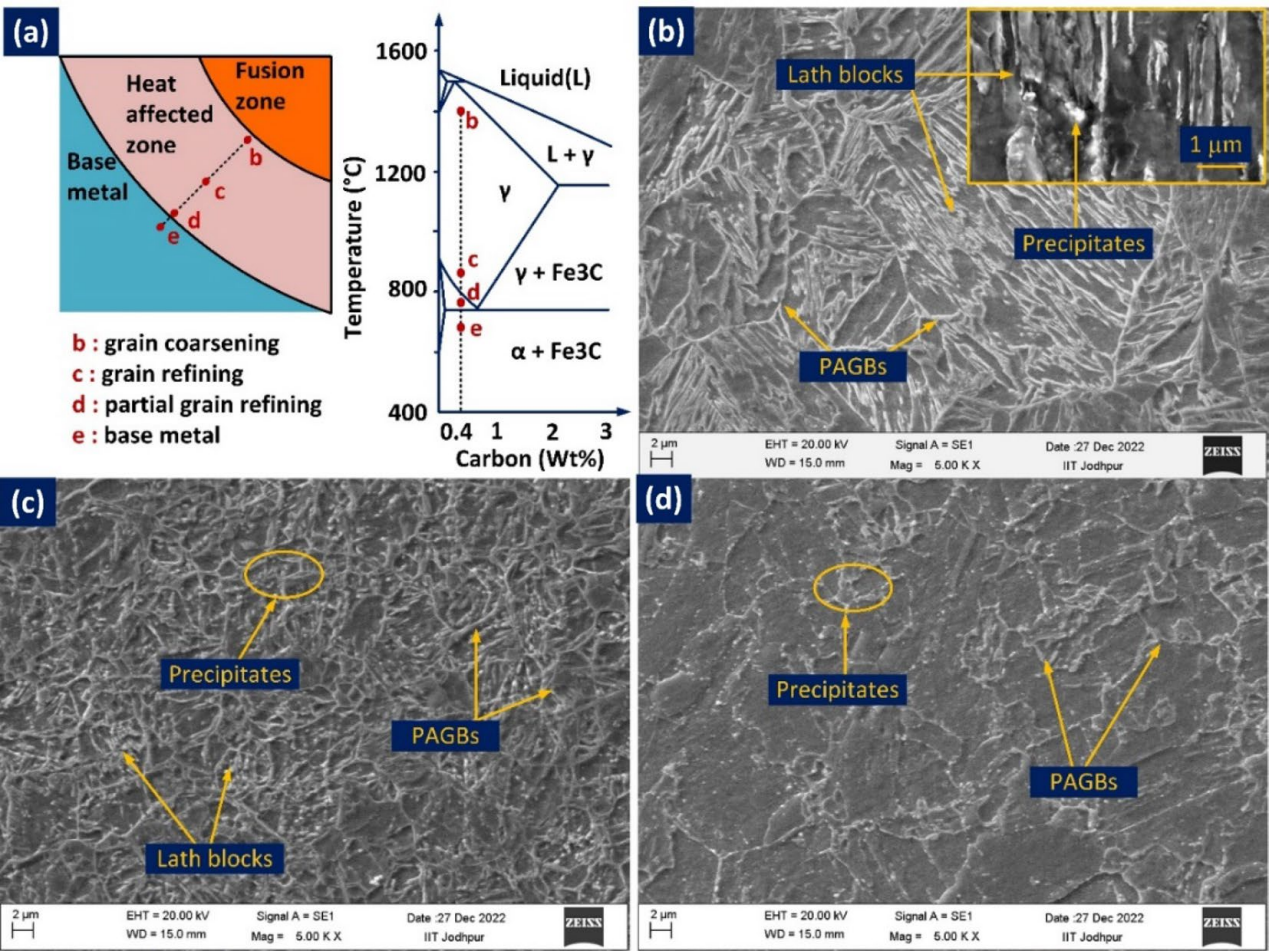
HAZs of P92 for NVG welds joint: (**a**) HAZs in schematic phase diagram showing each location and their corresponding temperature^60^ (**b**) coarse-grained HAZ (location b), (**c**) fine-grained HAZ (location c), (**d**) refined grains HAZ (RGHAZ (location d).

The HAZ of P92 BM is presented in Fig. 14. Based on peak temperature attained and grain size, HAZ is divided into three distinct regions, namely coarse-grained HAZ (CGHAZ) (location b), fine-grained HAZ (FGHAZ) (location c), and refined grains HAZ (RGHAZ) (location d), as displayed in Fig. 14a^60^. It is observed from Fig. 14a that CGHAZ is close to the FL and the peak temperature attained by this region is well above than A_c3_ temperature and allowing the precipitates to dissolve in the matrix and coarsening of the grains. Due to the high cooling rate, the coarse austenitic grains get transformed to martensite on cooling and exhibit the lath martensitic structure with well-defined lath blocks and boundaries as displayed in Fig. 14b. FGHAZ adjacent to CGHAZ is observed at a much lower temperature, i.e. just close to or above A_c3_. The low temperature limits the grain progress and on cooling, it transforms into a fine microstructure containing untempered martensite. The low temperature allows the partial dissolution of precipitates and the undissolved precipitates show a coarsening nature as given in Fig. 14c. In the end, the region of RGHAZ next to FGHAZ and adjacent to BM attains the peak temperature between A_c3_ and A_c1_. The temperature is not enough to convert the complete martensitic microstructure in austenite during heating which causes evolution of complex microstructure during cooling which contains both fresh martensite and over-tempered martensite. A negligible dissolution of the precipitates is also viewed from the SEM image presented in Fig. 14d.

**Figure 15:**
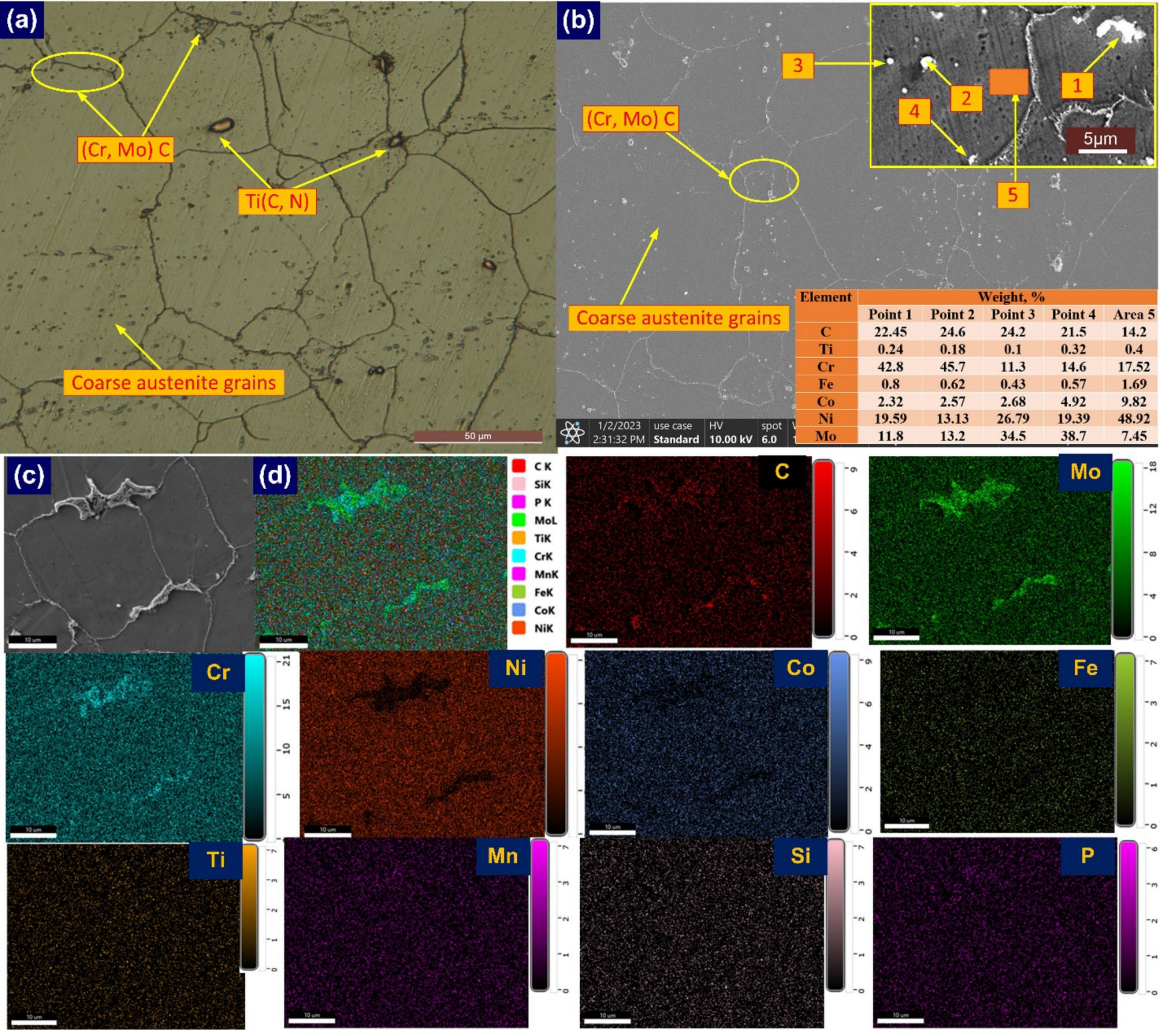
HAZ of Alloy 617 (**a**, **b**) optical and SEM (**c**, **d**) HAZ area selected for mapping and elemental area map.

The Alloy 617 HAZ does not show such microstructural features as observed for P92 however a grain growth is detected near FL (Fig. 15). In the optical study, an identical microstructure similar to Alloy 617 BM is predicted for HAZ for NVG welds joint (Fig. 15a). The coarse austenite grains, annealing twins and precipitates of different shapes and morphology are evident from the optical image. The average austenite grain size in HAZ was 113 ± 34 µm. The increase in grain size of HAZ compared to BM is the witness of grain growth. It is apparent from the SEM image that precipitates within the matrix are distributed in a random manner (Fig. 15b). The compositional analysis of precipitates is conducted using EDS point analysis and an elemental area map. The precipitates show the key concentration of the Cr, C and Mo in point EDS analysis and it could be the phase of Cr and Mo-rich M_23_C_6_ or Mo-rich M_6_C^61^. EDS of precipitates marked by arrows 1, and 2 shows that precipitates have a major weight percentage of Cr (point 1: 42.8%, and point 2: 45.7%), Mo (point 1: 11.8%, and point 2: 13.2%) and C (point 1: 22.45%, and point 2: 24.6%) and based on this study one can conclude that precipitates are Cr rich M_23_C_6_ phases. Ding et al.^61^ have also predicted the availability of the coarse and lamellar type of M_23_C_6_ carbide phases in Alloy 617 HAZ. The other fine precipitates available in the matrix are witnessed to be enriched with Mo, Cr and C (points 3: 34.5% Mo, 11.3% Cr and 24.2% C, point 4: 38.7% Mo, 14.6% Cr and 21.5% C) and it could be the possible phase of M_6_C enriched with Mo. The availability of the Ni, Co, Cr, Mo, Fe and Ti is witnessed from the EDS of the matrix (area 5). The austenite boundary with a higher concentration of Cr and precipitates with a greater concentration of Cr and Mo in the area elemental map are also evident of M_23_C_6_ and M_6_C phase formation in Alloy 617 HAZ (Fig. 15c, d). The availability of the Ti(C, N) in the Alloy 617 BM matrix has already been observed from EDS (Fig. 4) while, in Alloy 617 HAZ, no clue related to the Ti phase was seen in both EDS and area mapping. However, the phase of Ti(C, N) in the weld metal is evident from Fig. 11b.

#### Weld metal characterization

**Figure 16:**
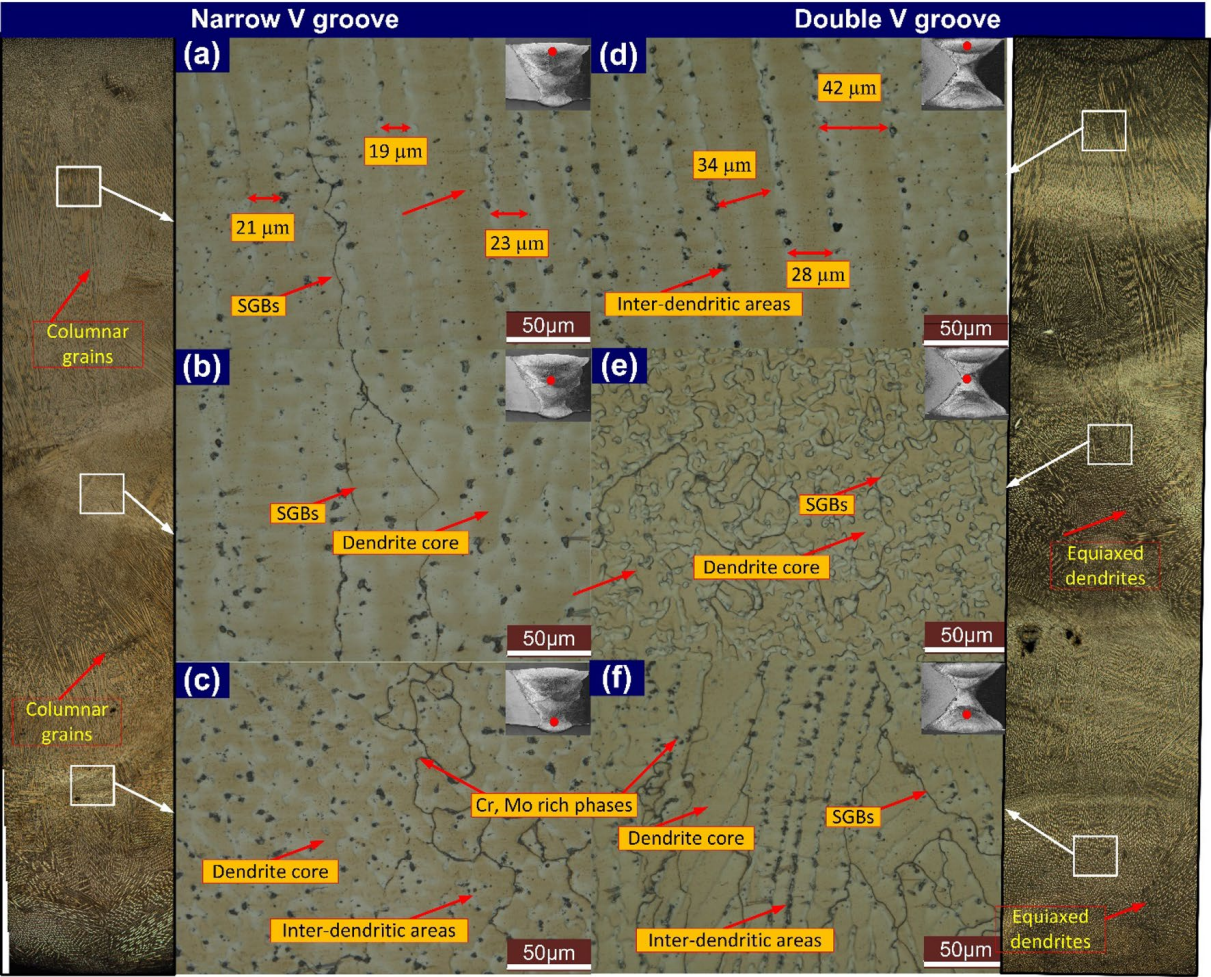
Weld metal optical image corresponding to capping (**a**), filling (**b**) and root (**c**) pass for NVG welds joint, capping (**d**), root (**e**) and filling (**f**) pass DVG welds joint.

To characterize the microstructure of ERNiCrCoMo-1 weld, the capping, filling and root pass regions are selected as mentioned in Fig. 16. The weld metal with distinct grain boundaries and dispersed phases are observed very clearly in Fig. 16. A clear and distinct dendritic microstructure with random orientation is observed from the optical image. The variation in microstructure from top to root is clearly distinguished from the optical image. However, compared to NVG welds joint, the major portion of the weld metal in DVG has equiaxed grains. In the root pass, the region of the weld adjacent to FL is subjected to a high cooling rate, which results in the formation of the columnar and cellular type of the grains. In the centre region of the weld corresponding to the root pass, the equiaxed types of grains are formed because of the slow cooling rate (Fig. 16c,e). In DVG, a major portion of the region contains the equiaxed type of grains however, in NVG, columnar and cellular grains are also observed in major areas. The effect of the welding heat from subsequent welding passes on microstructure evolution is also observed. As compared to capping and filling passes, the area of the weld metal corresponding to the root pass shows a higher density of the phases. Figure 16b,f displays the microstructure corresponding to the filling pass. The region also experiences a high cooling rate due to its thin nature and the presence of the weld metal in the root pass. However, the annealing of the weld metal in the filling pass region also occurs due to the reheating from the capping pass. The weld metal adjacent to FL has columnar dendritic and cellular grains however they differ from each other in terms of segregation and grain morphology, and it could be due to the different composition and cooling rates on both sides of FL. Different grain morphology of the weld metal is seen for both the welds joint corresponding to the filling pass. In NVG, weld metal shows the columnar grain however, in DVG welds joint, an equiaxed dendritic microstructure is observed. In the capping pass region, a typical dendritic microstructure of the columnar dendrites is observed (Fig. 16a,d). The arm spacing was 21 µm and 35 µm for NVG and DVG welds joints.

**Figure 17:**
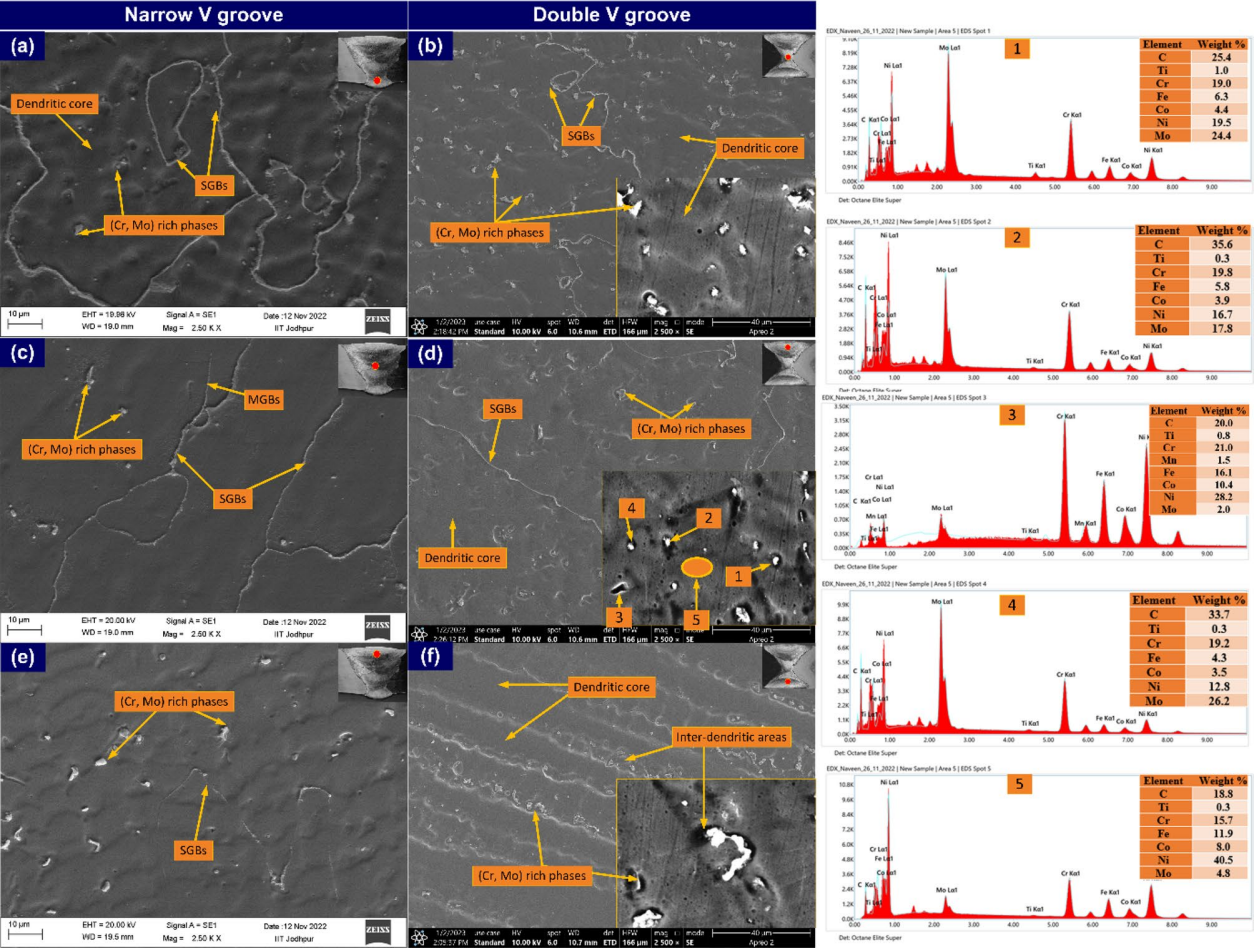
Weld metal SEM image for both joints captured at a different location: (**a**, **b**, **c**) NVG welds joint, (**d**, **e**, **f**) DVG welds joint, EDS point analysis of different location marked in (**d**).

To observe the morphology of the precipitates and their distribution in the matrix, an SEM image is also captured for different regions of the weld metal, including capping, filling and root pass area. Like optical, SEM image also confirms the equiaxed structure in the root area, possibly due to the annealing effect of the subsequent welding pass. The precipitates are distributed uniformly within the matrix and mainly encountered along the inter-dendritic areas and SGBs (Fig. 17a,b). The SEM image of weld metal corresponding to the filling pass shows columnar grains for NVG and cellular type of the grains for DVG welds joint. That could be possible due to the different cooling rates (Fig. 17c,d). However, compared to root and capping passes, the density of the precipitates is observed to be less for weld metal corresponding to the filling pass. The weld metal corresponding to the filling pass have cellular grains for NVG welds joint and typical columnar dendrites for DVG welds joint and it occurs due to a high cooling rate as it is directly exposed to the surrounding. The variation in precipitate density and morphology is seen clearly in the SEM image. The grain boundaries in the capping pass region of NVG welds joint are not visible and it might be due to the non-existence of the precipitates (Fig. 17e). A decreasing trend of a fraction of precipitates from root pass to capping pass is observed, which could be due to the reduced annealing effect from subsequent passes as well as enhanced cooling rate as discussed in optical micrographs. The EDS point analysis is carried out for white particles and dendrite core present in the weld metal of the DVG welds joint as marked in Fig. 17d. The EDS results of the white particles indicated by point 1, point 2, and point 4 confirm the major concentration of the Mo and C (point 1: 24.4% Mo and 25.4% C; point 2: 17.8% Mo and 35.6% C; point 4: 26.2% Mo and 33.7% C) and the particles could be the Mo_6_C phase which is also detected in the previous study^62^. The Cr (21.0%) and C (20.0%) are observed as major elements in the EDS of the grey particles marked as point 3. This certified that the grey particle present in weld metal could be the possible phase of Cr-rich M_23_C_6_. The concentration of the Mo and Cr in inter-dendritic areas is measured lower than the dendrite core area marked by arrow 5 in Fig. 17d. The weight percentage of Mo (point 4) and Cr (point 3) in secondary phases is increased by 445% and 22%, compared to the dendrite core (marked by arrow 5). The depletion of the core from Mo could be observed easily from EDS and it can be affirmed that phases present along the inter-dendritic areas are Mo_6_C and M_23_C_6_. The existence of these carbide phases imparts the hardening effect to weld metal however its negative impact on Charpy toughness is well known. It is known that in Ni-based filler alloying elements like Mo and Nb are added to enhance the strength and hardness of the welded joint however its severe segregation in inter-dendritic liquids during the solidification results in the establishment of the various type of carbide and inter-metallic phases of complex geometry (M_23_C_6_, M_6_C, MC, laves phase) which reduces the mechanical performance of the welded joint^14,63^. The segregation of the alloying elements during the solidification is mainly explained by the partition coefficient (k). According to a study by Naffakh et al.^25^, elements with k values lower than 1 are more likely to redistribute during the solidification stage. The element composition at front of the liquid/solid interface varies during the solidification process because of the elemental redistribution. The k is used to define the elemental redistribution and calculated by the relation (k) = Cs/Co, where Cs and Co present the concentration at the dendrite core and nominal composition in the weld. The k value of different elements was evaluated and it was 0.52 and 0.89 for Mo and Cr, respectively. It was reported that an element with a k value close to 1 (Cr = 0.89) shows an equal tendency for segregation in both solid and liquid. However, the element with a k value much lower than 1 (Mo = 0.52) showed a strong segregation tendency into the inter-dendritic liquids during the solidification. In ERNiCrCoMo-1 weld metal, the solidification process starts from the primary reaction L → ϒ (austenite) and by which Mo separates from the ϒ dendrites/solidification front and comes into inter-dendritic liquid^64^. As solidification progresses, the Mo concentration in ϒ dendrites increases at the solid/liquid interface and after a certain period of time, it reaches the maximum solid solubility level in austenite at the edge of the ϒ dendrites. The poor diffusion rate of Mo in austenite also helps to retard their back diffusion in dendrite and develops a stable concentration gradient. In the late solidification stage, Mo get combines with C and produces the Mo-rich M_6_C phases. The solubility of Mo in the austenite matrix can be reduced by increasing the Fe content. However, in such cases, Mo segregated at boundaries gets combined with Fe and promotes the formation of the intermetallic Laves phase (Fe_2_Mo). The large radii of Mo compared to matrix element Ni, also increase their segregation intensity and it get rejected into inter-dendritic locations during solidification and enriches it with phases of Mo. However, a small amount of the Cr rich M_23_C_6_ phase (point 3) is detected in point EDS analysis which was grey in colour. The previous report also confirmed the presence of Cr rich phases in inter-dendritic areas due to the rejection of Cr from dendrite core to inter-dendritic boundaries^65^.

**Figure 18:**
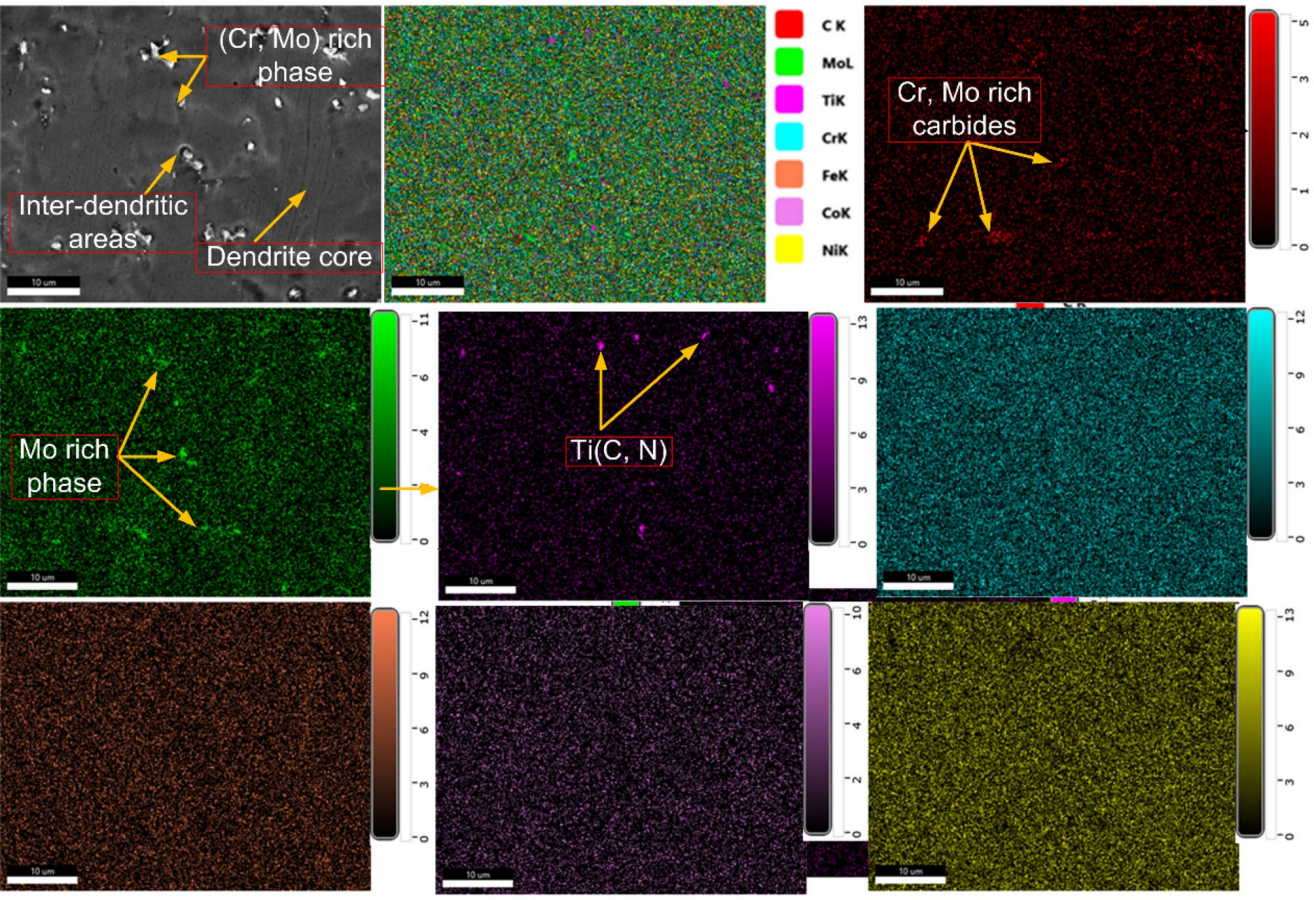
Elemental map confirming the presence of Cr, Mo and Ti-rich phases in the weld metal.

**Figure 19:**
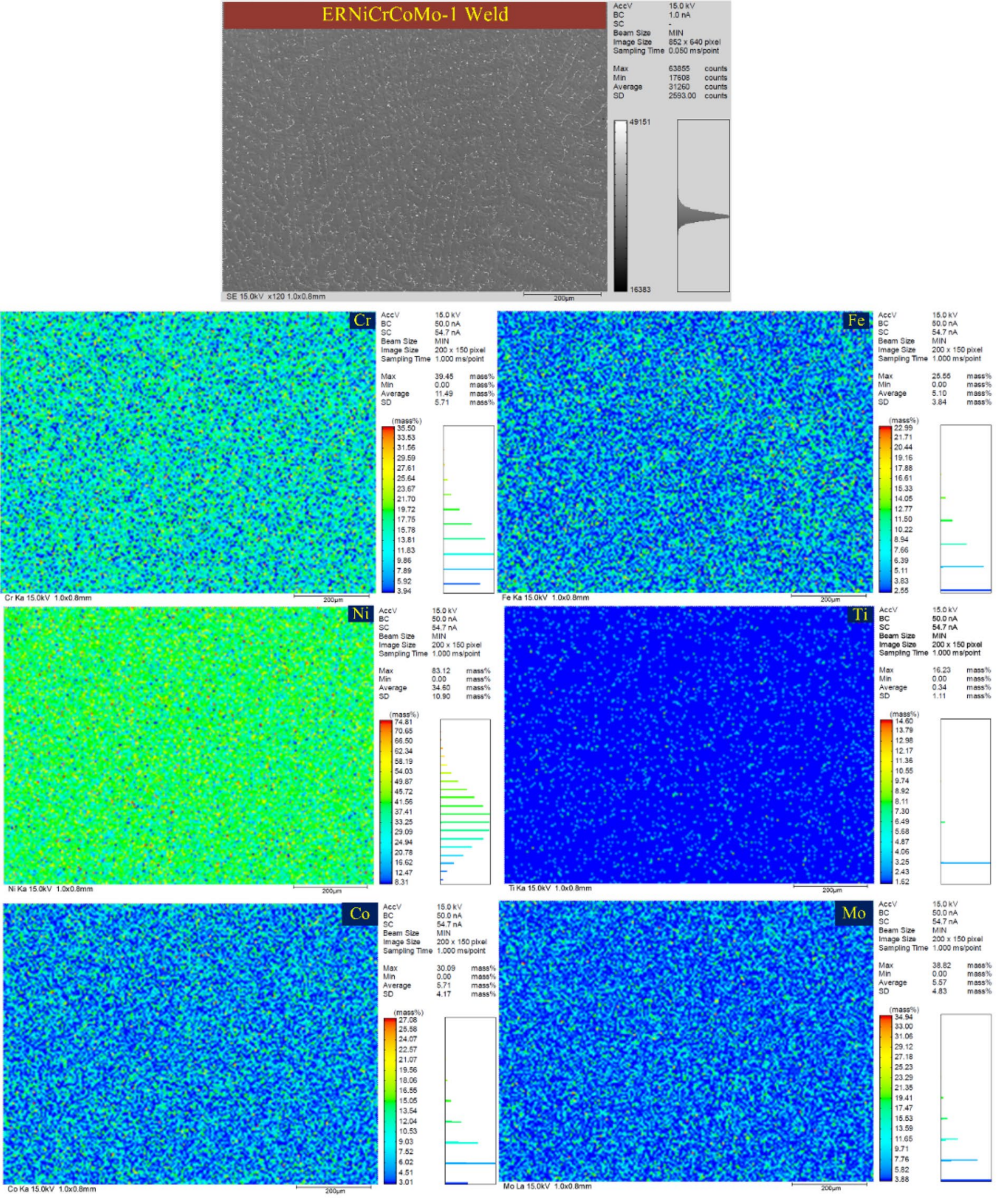
EPMA of the ERNiCRCoMo-1 weld showing the concentration of various elements along the dendrite core and boundaries.

The SEM/EDS map of the ERNiCrCoMo-1 weld (presented in grey colour) is displayed in Fig. 18. It is evident from the map that inter-dendritic regions have a major concentration of Mo. Fe and Co show an almost uniform distribution. However, Cr concentration at a few white particles is observed higher than other elements. The Ti concentration is observed higher at a few intra-granular areas and also at a few particles located at boundaries. From the complete map, it can be concluded that Mo-rich phases are mainly present in inter-dendritic areas which is also been confirmed from EDS point analysis. The Ti-rich phases are present randomly in intra-granular areas at boundaries. Due to higher Cr concentration, Cr-rich M_23_C_6_ phases are also expected in a few regions of boundaries. The micrograph of ERNiCrCoMo-1 weld in grey colour shows the austenitic grains with white-coloured low melting eutectics along the boundaries (Fig. 19). The presence of the Mo-rich secondary phases are inferred from the EPMA-based mapping. The Ti-rich phases in intra-granular areas and also along the boundaries are witnessed from the EPMA map. The same has also been confirmed by the SEM/EDS mapping presented in Fig. 17.

### Mechanical testing

#### Microhardness of weldment

**Figure 20:**
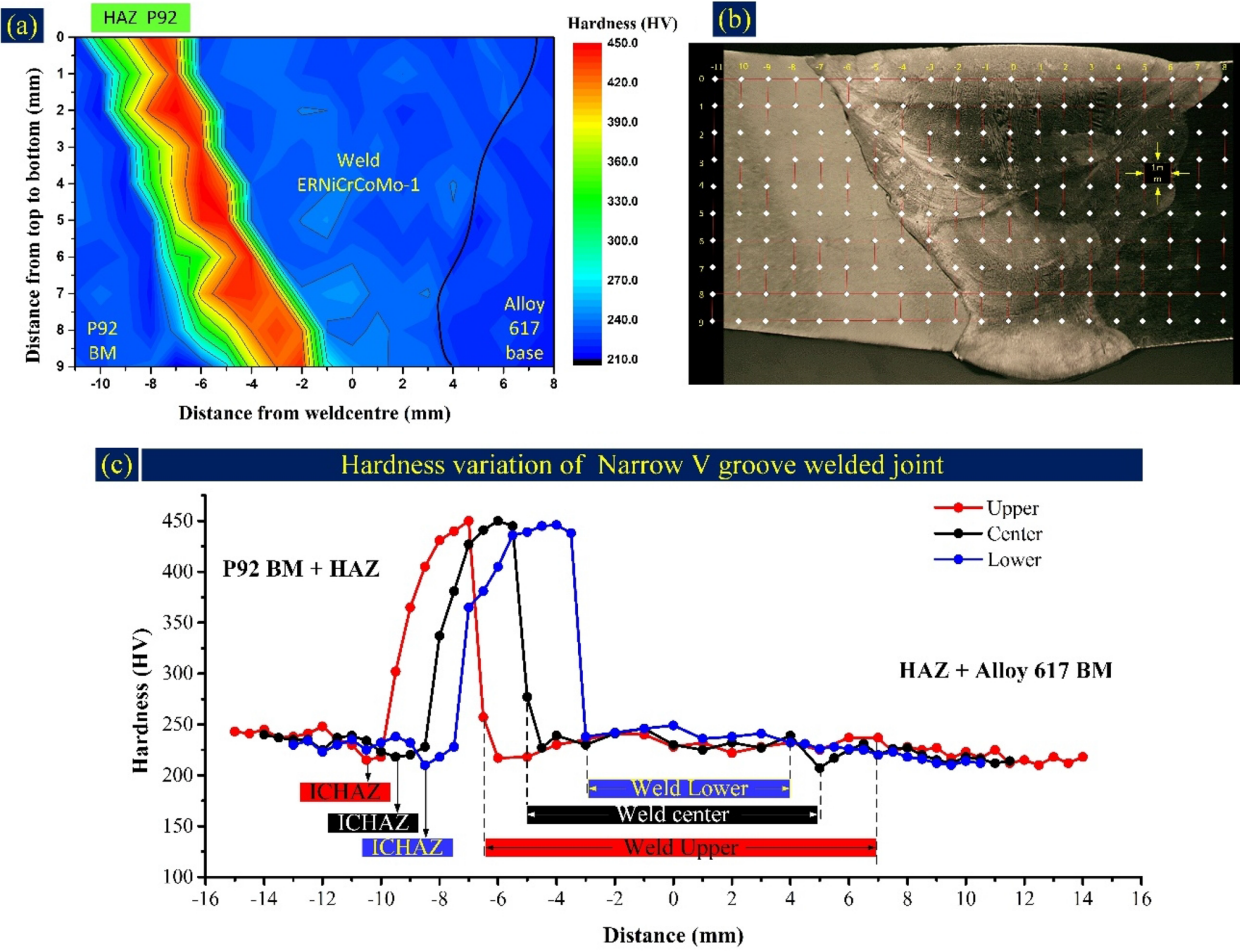
Microhardness of NVG weld joint, (**a**) Microhardness map of weld joint cross-section, (**b**) indent matrix location on the weld joint cross-section, (**c**) hardness variation profile at the upper (top), center, and lower (root) section of weld joint cross-section.

**Figure 21:**
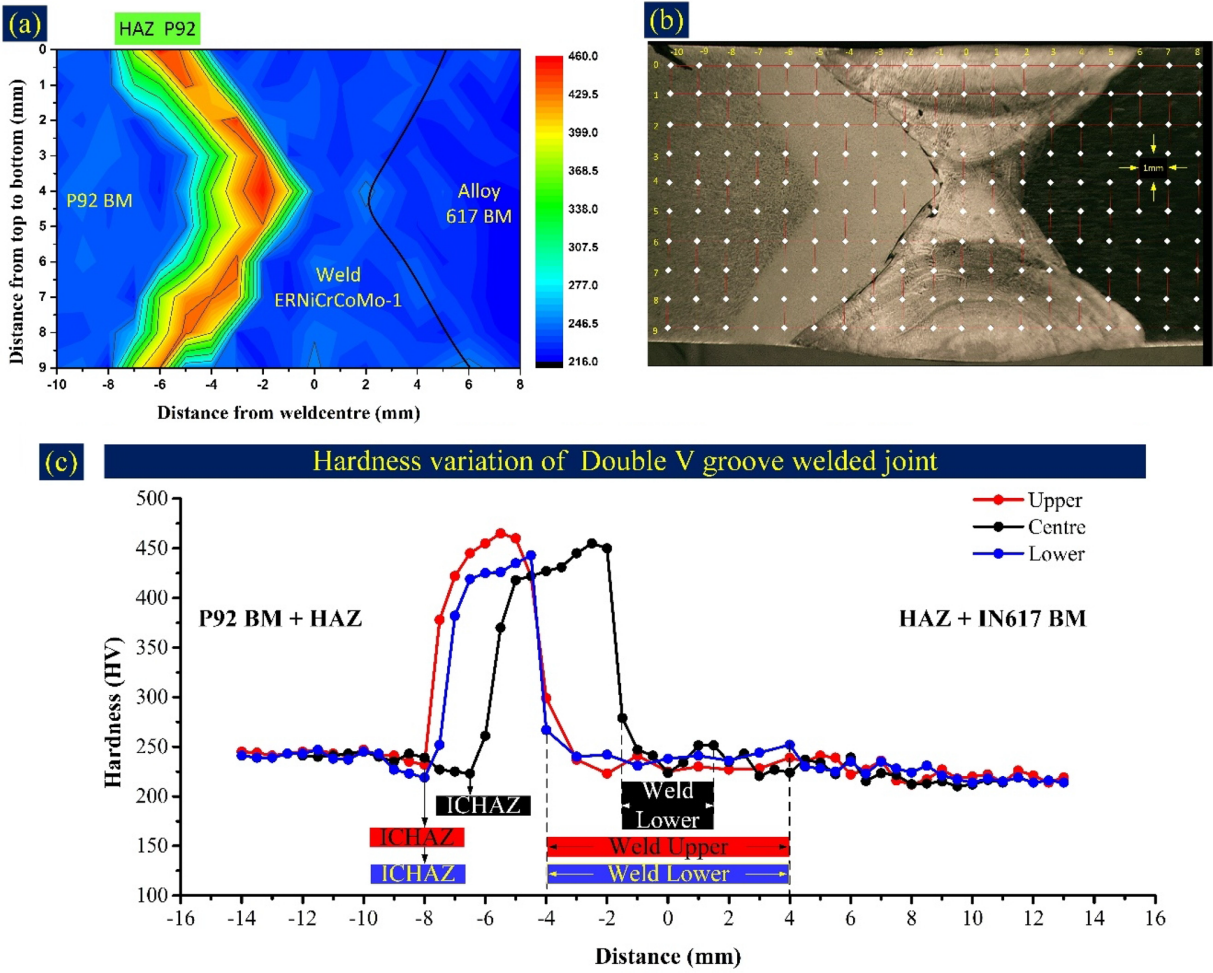
Microhardness of DVG weld joint, (**a**) Microhardness map of weld joint cross-section, (**b**) indent matrix location on the weld joint cross-section, (**c**) hardness variation profile at the upper (top), centre (root), and lower (filling) section of weld joint cross-section.

The hardness indent was taken in the transverse direction corresponding to each welding pass, as mentioned in Figs. 20, 21. The Matlab map is also plotted for both NVG and DVG welds joint and each indent position is marked on the macrograph (Figs. 20b, 21b). The cross-section area of 18 mm × 9 mm was selected for hardness measurement at a gap of 1 mm. Based on the measured data, a map was plotted for both the welds joints, which quantifies the material quality of each region of the weldments (Figs. 20a, 21a). The mapping aimed to pinpoint the precise hardness differential all over the weldment and deliver reliable data. The hardness plot includes all the zone of weldments for three different passes (capping, filling and root pass), as mentioned in Figs. 20c and 21c for NVG and DVG welds joints, respectively. The average hardness of the weld zone, HAZ and interface corresponding to each welding pass are mentioned in Table 2. The hardness of weld metal was 229 ± 8 HV, 233 ± 7 HV and 242 ± 5 HV for capping, filling and root pass, respectively for NVG welds joint. For DVG welds joint, it was 230 ± 7 HV, 242 ± 11 HV and 239 ± 4 for capping, root and filling pass, respectively. The peak hardness in weld metal was 249 HV, corresponding to the root pass in NVG welds joint, and 251 HV, corresponding to the root pass in DVG welds joint. Hence, it could be stated that the peak hardness of weld metal was in the root pass for both the welds joint. In the root pass, the redistribution of the phases and higher density of the phases as a result of the reheating effect of other passes contribute to the higher hardness to weld metal corresponding to the root pass compared to other passes. The higher density of the carbide phases (mainly Mo_6_C) comes from the higher Mo concentration in weld metal corresponding to the root pass. The Mo-rich phases increase the precipitation strengthening and provide the hardening effect. The higher density of the Mo-rich secondary phases also provides the hardening effect to the weld zone that reflects in hardness results. The large atomic size of Mo compared to other alloying elements of the filler also produces a high amount of distortion in the matrix lattices, which results in an increased hardness^66^. The other possible cause might be that the root experiences a slower cooling rate after the first pass, leading to a dendritic structure that is more refined than the capping and filling pass. Evidence also suggests that the root undergoes plastic deformation due to the temperature gradient between the capping pass and the root pass^9^. However, the variation in hardness corresponding to each pass along the transverse direction is governed by both carbide phases as well as the microstructure (columnar/cellular/equiaxed). The variation in microstructure along the transverse direction, i.e. from FL to the centre of weld metal has already been discussed in the metallographic section. Compared to Alloy 617 BM (217 ± 4 HV), the high hardness of the weld metal could be attributed to the solid solution strengthening elements like Co, Cr and Mo in filler metal. The elements like Mo having limited solubility in the austenitic matrix also enrich the matrix with Mo-rich carbide phases as evident from the SEM/EDS of weld metal and increase the hardness by precipitation hardening effect. The hardness of Alloy 617 HAZ was between 223 ± 4 and 225 ± 4 HV for NVG welds joint and 227 ± 6 HV to 231 ± 4 HV for DVG welds joint, respectively. The hardness value of HAZ was measured lower than the Alloy 617 BM and it could be due to the higher dislocation density and availability of lamellar and coarse carbide phases of Cr and Mo^61^. The microstructure difference between Alloy 617 HAZ and BM was also unobvious as discussed in the metallographic section. A dramatic variation in the hardness of P92 HAZ is observed, and the weldments’ peak hardness is also measured in CGHAZ of P92 steel. The CGHAZ hardness corresponding to each pass is mentioned in Table 2. It was in the range of 442 HV-448 HV for NVG welds joint and 432 HV-450 HV for DVG welds joint. The peak hardness to CGHAZ is attributed to the solid solution hardening that was obtained due to the dissolution of the precipitates in the matrix during welding^67^. The widest region among P92 HAZs was FGHAZ and temperature also varied in this region from peak temperature above than or close to A_c3_ adjacent to CGHAZ to temperature just above A_c1,_ adjacent to ICHAZ. That results in great variation in the hardness of the FGHAZ. In NVG welds joint, the ICHAZ was recognized as the weakest zone in terms of hardness and its hardness was between 212 and 218 HV. The hardness of ICHAZ was estimated much lower than the average hardness of CGHAZ/FGHAZ and BM (239 ± 6 HV) of P92. The same variation is observed for DVG welds joint however, hardness of ICHAZ was 219–232 HV which was lower among all the zones except Alloy 617 BM (217 ± 4 HV). The complex microstructure and availability of the soft ferrite could be the possible cause of the poor hardness of ICHAZ. The major problem of the dissimilar welded joint is the formation of the UZ. Hence it becomes important to measure their hardness. The results of interface hardness measurement are given in Table 2. The considerable width of the UZ was observed near the P92 interface. The hardness at P92 interface was measured 257 HV, 277 HV and 238 HV for capping, filling and root pass, respectively in NVG welds joint. For capping and filling pass, the hardness at the interface was observed higher than the weld metal and P92 BM, which might be due to the availability of the Cr, Co, Ni and Mo, obtained due to the diffusion from weld metal. The hardness of the interface near to root pass was measured close to the hardness of P92 BM and weld metal. At Alloy 617 interface, hardness was 212–237 HV, close to the weld metal and Alloy 617 BM hardness. From the interface hardness of Alloy 617, it can be inferred that there is a high dilution of the filler due to the negligible variation in the composition and melting point of both Alloy 617 BM and filler. A similar observation has also been made for DVG welds joint. From the hardness indent plot, it is clear that there is variation in hardness both along the transverse and longitudinal direction however, groove geometry has seen a negligible or minor effect on the hardness plot and hardness value of different zones.

**Table 2:** Hardness of distinct zone of weldments.

	Narrow V groove welds joint			Double V groove welds joint		
Hardness (HV)_0.5_	Capping	Filling	Root	Capping	Root	Filling
Average hardness of weld metal	229 ± 8	233 ± 7	242 ± 5	230 ± 7	242 ± 11	239 ± 4
P92 HAZ (CGHAZ/FGHAZ/ICHAZ)	445 ± 4/344 ± 86/215	448 ± 6/340 ± 96/218	442 ± 3/385 ± 78/212	432 ± 4/337 ± 32/232	450 ± 2/404 ± 63/223	443 ± 3/398 ± 67/219
Interface (P92/Alloy 617)	257/237	277/212	238/232	229/239	279/251	267/252
IN617 HAZ	223 ± 4	224 ± 5	225 ± 4	227 ± 6	231 ± 4	230 ± 5
Peak hardness of weld metal	241	246	249	241	251	244
P92 steel	239 ± 6					
Alloy 617	217 ± 4					

#### Impact energy of weldment

**Figure 22:**
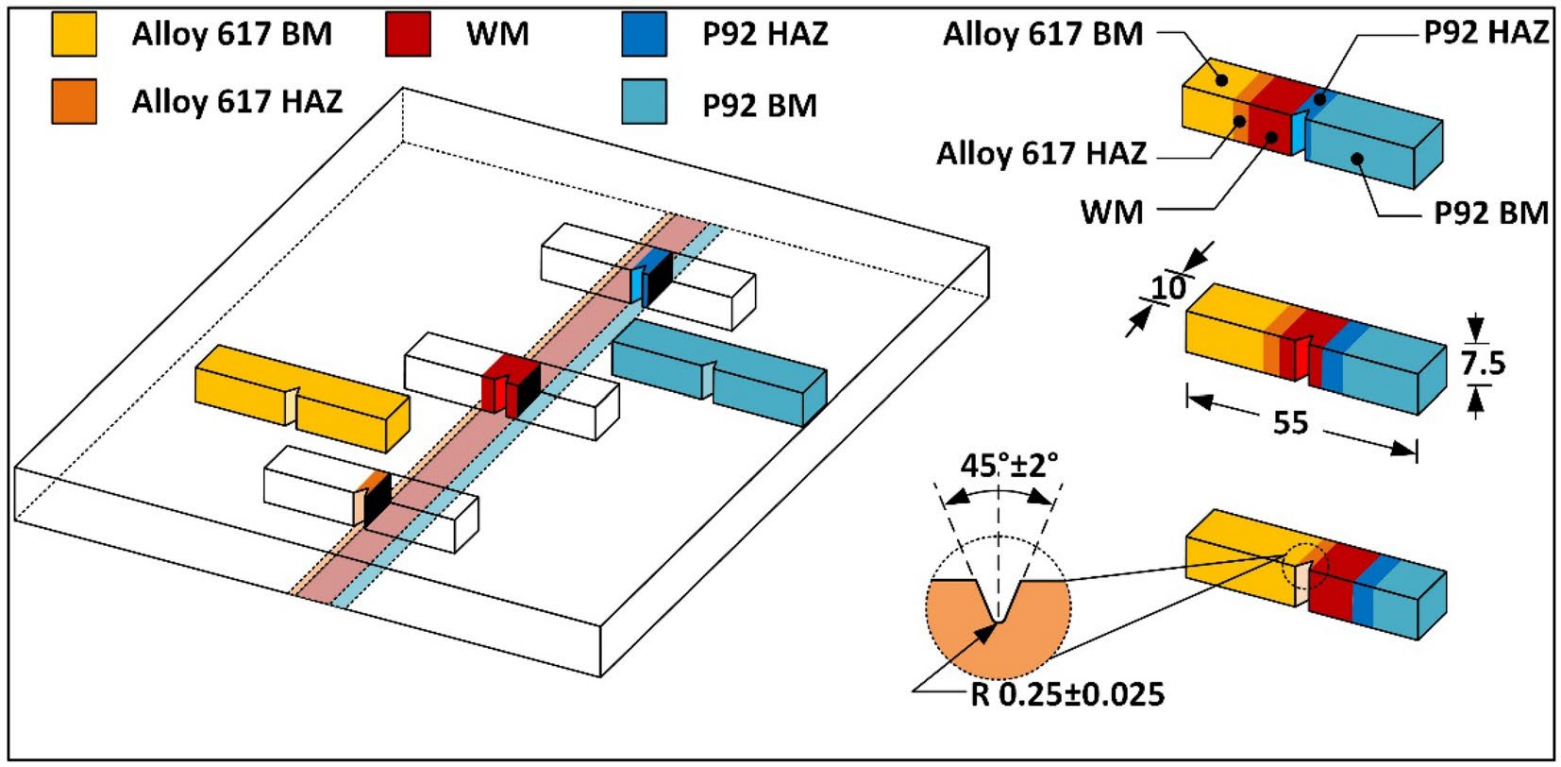
Charpy impact specimen preparation from a different region of the weldments.

**Figure 23:**
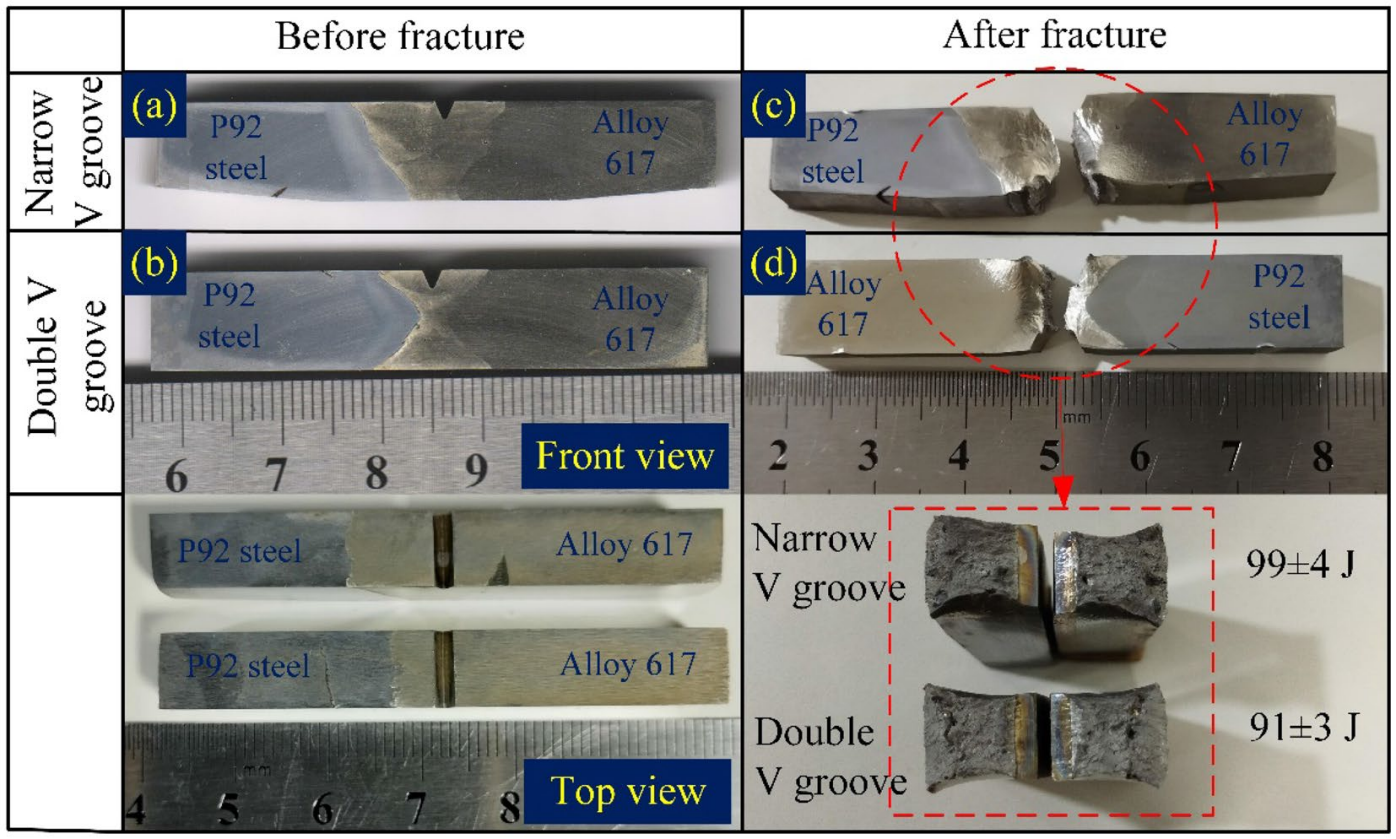
Specimen of Charpy impact testing for and NVG and DVG joint (**a**) front view, (**b**) top view, (**c**) after testing, (**d**) top view after testing and test results.

The samples of Charpy impact specimen were machined using wire cut EDM to investigate the energy absorbing capacity of the different regions of the weldments during impact loading (Fig. 22). The detail of the notch location and their dimensions are mentioned in Fig. 22. The specimen of the weld metal for both NVG and DVG joints is displayed in Fig. 23. The front and top view of the weld metal impact specimen before fracture is displayed in Fig. 23a,b. P92 and Alloy 617 BMs have an average impact energy of 150 ± 4 J, and 142 ± 3 J, respectively (Table 3). For both NVG and DVG joints, specimens were braked in two parts with a small amount of plastic deformation as given in Fig. 23c. The impact energy of the NVG joint was 99 ± 4 J which was very close to the impact energy of the DVG joint (91 ± 3 J). The top surface of the fractured specimen is displayed in Fig. 23d. According to the European Standard EN ISO15614-1:2017, 47 J is the minimum impact energy required to satisfy the requirements for boiler and piping applications and to limit the likelihood of brittle failure in the weld metal^52^. However, for qualification of the dissimilar joint under fast breeder reactor application, the minimum acclaimed value of the impact energy is 80 J^68^. Thus, weld metals for both NVG and DVG joints meet the criteria for boiler applications. However, a considerable drop in impact energy of the weld metal as compared to BMs was inferred from the test results. During the welding process, the inter-dendritic regions of the weld get enriched by the Cr, Ti, C and Mo, present in Alloy 617 filler metal which led to the formation of the brittle carbide phases which was one the possible causes of reduction in impact energy value. A measurable effect of the hard secondary phases and inter-metallic phases of weld metal on the impact energy of the Ni-based welded joint has already been reported^59^. The dendritic microstructure and element segregation along the inter-dendritic spaces of the weld metal in the Ni-based filler primarily control their impact energy^53^. Dupont et al.^5^ also observed that secondary phases affect weld metal impact energy in Ni-based alloys. The higher density of these hard phases (Cr and Mo rich) provides the dense microcrack during the impact testing. This results in poor impact resisting capacity of the weld metal than the BMs. The results obtained for NVG and DVG joints were close to the previously published report (98 ± 5 J) of Kumar and Pandey^69^ where a conventional V groove joint was manufactured for the same plate and filler metal. The impact energy of Alloy 617 HAZ (122 ± 3 J: NVG joint and 128 ± 4 J: DVG joint) and P92 HAZ (128 ± 6 J: NVG joint and 135 ± 5 J: DVG joint) was estimated inferior to the BM but greater than the weld metal. The untempered martensitic microstructure in the coarse-grained HAZ area reduced the impact energy of the P92 HAZ lower than the BM^70^ while a drop in impact energy of IN617 HAZ was possibly caused by coarsening or dissolution of the carbide phases^59^. Ding et al.^61^ also observed the reduction in impact energy of Alloy 617 HAZ due to the availability of lamellar carbide layer and coarse carbides at grain boundaries.

**Table 3:** Tensile properties of the welded joint and BMs at room temperature and 650 °C.

		Room temperature				High temperature (650 °C)		
		P92 steel BM	Alloy 617 BM	Narrow V groove WJ	Double V groove WJ	P92 Steel BM	Narrow V groove WJ	Double V groove WJ
Tensile Properties	Ultimate tensile strength (MPa)	760 ± 4	775 ± 7	748 + 2	696 ± 5	318 ± 2	278 ± 3	275 ± 2
	Mismatch factor (WMYS/BMYS)	–	–	0.98/0.97 (P92/Alloy 617)	0.91/0.90 (P92/Alloy 617)	–	0.88 (P92)	0.88 (P92)
	Joint efficiency (%)^71^	–	–	97	90	–	–	–
	Fracture location	–	–	P92 BM	P92 BM	–	P92 BM	P92 BM
Charpy toughness		150 ± 4 J	142 ± 3 J	99 ± 2	91 ± 4			

**Figure 24:**
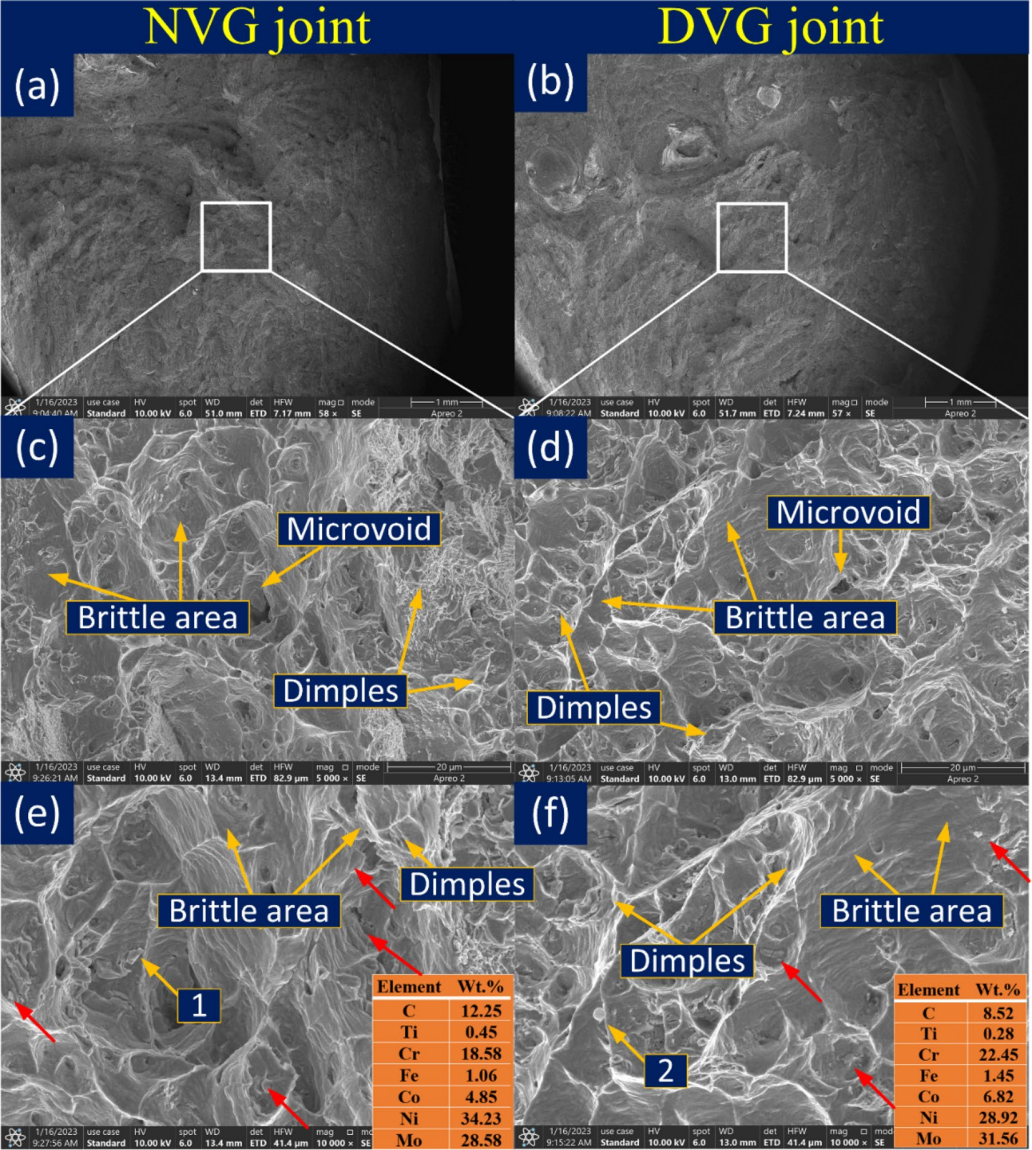
Fracture surface characteristic of impact tested specimen (**a**, **c** and **e**) NVG joint (**b**, **d** and **f**) DVG joint.

The fracture surface after impact loading is characterized using SEM and a different view of the same is displayed in Fig. 24. The top view is given in Fig. 24a,b for NVG and DVG joints, respectively. For both joints, mixed more of the fracture is witnessed which exhibits microvoids, dimples and brittle areas. Between NVG and DVG, the former one shows fine dimples, negligible voids and large brittle (Fig. 24c) areas while the later one displays shallow dimples, microvoids of larger in quantity, the shear plane with dimples facets and brittle areas (Fig. 24d). As observed from the impact testing results that weld metal has poor impact energy in both the joints and one possible cause of this could be the enrichment of the inter-dendritic boundaries of the weld metal with Mo rich phases. Mo-rich phases are also evident from the detailed view of the fracture surface and EDS results (Fig. 24e,f). The brittle Mo-rich phase (M_6_C) is mainly considered the source of stress intensity and during impact loading facilitates the nucleation of the microvoids. The crack propagation is mainly expected at the interface of the matrix and precipitates. The Mo and Cr weight percentage on fracture surface particles are 28.58% and 18.58% for NVG and 31.56% and 22.45% for DVG, respectively, as illustrated in Fig. 24e,f. The Cr and Mo-rich phases are mainly embedded within the shallow dimples. However, it is impossible to justify the poor impact energy value of the weld metal only on the basis of the fracture surface characteristic and Mo-rich phases because the mechanical properties (impact energy) of the material are also governed by the welding cycle (residual and thermal stresses) and phases transition under the influence of the welding cycle.

#### Tensile testing

**Figure 25:**
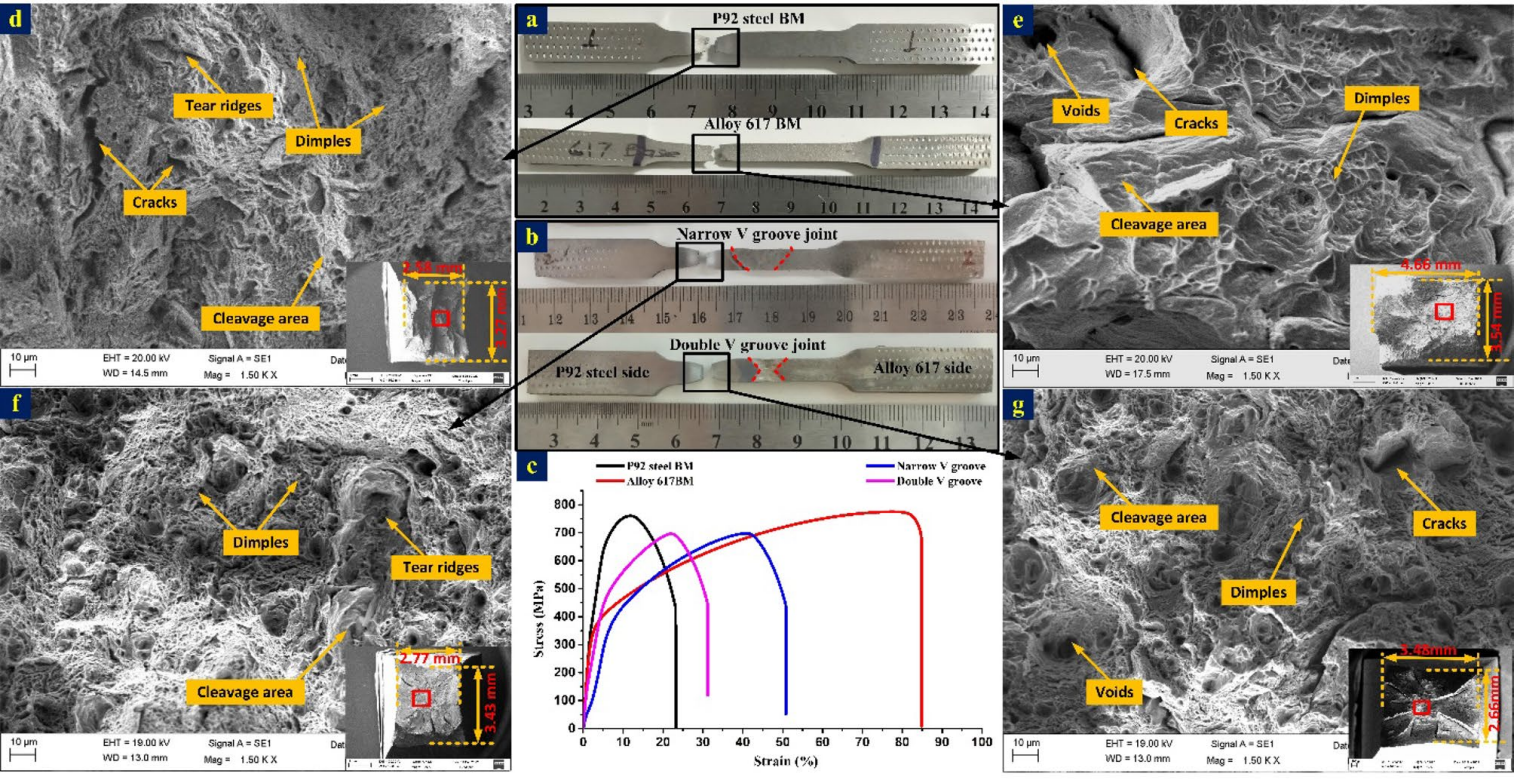
Macrograph of the ruptured tensile specimens: (**a**) BM-RT, (**b**) welded joint-RT; (**c**) stress–strain curve of joint and BMs; fracture surface image of (**d**) P92BM-RT, (**e**) IN617BM-RT, (**f**) NVG-RT, (**g**) DVG-RT.

The room temperature and high-temperature (650 °C) tensile specimens were prepared and tested for both welds groove joints and also for BMs. To simplify the discussion, the specimen of Alloy 617 BM and P92 BM tested at room temperature during uniaxial tensile tests were denoted by IN617BM-RT and P92BM-RT, respectively while welded joints tested at room temperature were denoted by NVG-RT and DVG-RT for NVG and DVG joints, respectively. In a similar fashion, high-temperature tensile tested specimens of P92 BM and welded joints were denoted by P92BM-HT, NVG-HT and DVG-HT. The macrograph of the ruptured tensile specimen of IN617BM-RT, P92BM-RT, NVG-RT and DVG-RT and their corresponding stress–strain curve is mentioned in Fig. 25a–c. The quantitative tensile test is mentioned in Table 3**.** The BMs properties were included to make a comparison with welded joints. The ultimate tensile strength (UTS) was 775 ± 6 MPa for IN617BM-RT and 760 ± 4 MPa for P92BM-RT. The NVG-RT and DVG-RT joint failed in the weaker parent metal, i.e. P92 BM which was similar to the previously reported work of Kumar and Pandey^69^. However, as per the hardness report, failure was expected in Alloy 617 as it offers poor hardness among all the zone. Also, the failure from the parent metal rather than the weld metal ensured the applicability of the welded joint for AUSC plants. The UTS was 748 + 2 MPa for NVG-RT and 696 ± 5 MPa for DVG-RT. The UTS of the NVG-RT and DVG-RT joints were measured lower than the BMs. The failure location was much away from the weldments as seen from the macrograph (Fig. 25b). The typical cup-cone fracture with neck formation at the fracture tip is observed in Fig. 25b. From tensile test results, it can be inferred that groove geometry has a negligible effect on tensile properties. The mismatch factor and joint efficiency were also estimated for both joints and presented in Table 3. For NVG welds joint, weld mismatch with P92 steel and Alloy 617 were 0.98 and 0.97 along with joint efficiency of 97%. For DVG welds joint, weld mismatch with P92 steel and Alloy 617 were 0.91 and 0.90, respectively, along with joint efficiency of 90%. The SEM image of the fractured P92 BM shows the dimples, cracks, voids and tear ridges in the major area while besides the dimples and voids cleavage area is also seen (Fig. 25d). The fracture surface of alloy 617 exhibits the cleavage facets in the major area (Fig. 25e) while cracks and shallow dimples are also seen besides the cleavage area. The fracture surface was also studied for NVG-RT and DVG-RT at low and high magnification. The top view of the fracture surface exhibited multiple cracks in both NVG-RT (Fig. 25f) and DVG-RT (Fig. 25g) characterized specimens. The magnified view exhibited dimples and voids of varying size and shape along with cleavage plane and tear ridges. However, a major portion of the fracture surface is governed by the fine dimples and voids which confirmed the dimples-dominated mixed mode fracture, as presented in Fig. 25f,g. The previous tensile test study of a conventional V groove welded joint of P92/Alloy 617 alloy produced with ERNiCrCoMo-1 also confirmed the failure from P92 BM however UTS was measured as 725 MPa^69^. In another study of similar welded joints produced using matching P92 filler, the UTS was measured in the range of 602–704 MPa with varying fracture locations, i.e. either in P92 BM or from the P92 interface^72^. For a similar type of joint produced using the radiant beam welding process, the UTS was measured in the range of 704–712 MPa with a fracture location of P92 BM^73^. Hence, the current study of the RT tensile test showed good agreement with the previous studies.

**Figure 26:**
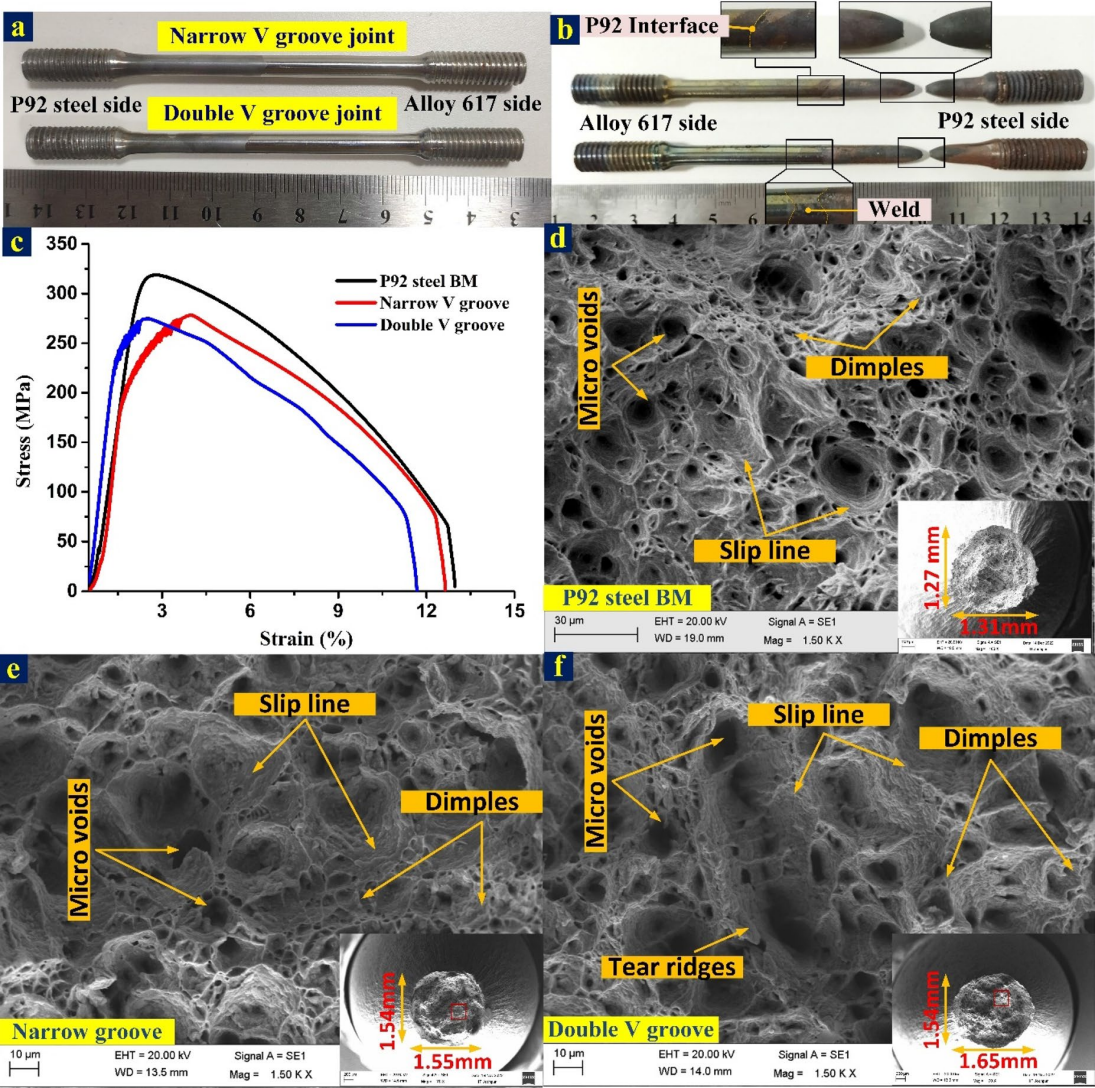
Macrograph of round tensile specimens of welded joint for the high-temperature tensile test (**a**) before rupture, and (**b**) after rupture, (**c**) stress–strain characteristic of welded joint and P92 BM; a top and detailed view of the fracture surface (**d**) P92BM-HT, (**e**) NVG-HT, (**f**) DVG-HT.

The round specimen was tested during a high-temperature tensile test which was conducted at 650 °C (Fig. 26a). The test results showed a significant drop in YS and UTS for both NVG-HT and DVG-HT as compared to BMs (Table 3). The results might be due to the softening effect and also because of the increased sensitivity to damage at high temperatures. However similar to the RT test, all the specimens failed from the P92 BM (Fig. 26b). The stress–strain plot is mentioned in Fig. 26c. From failure in P92 BM, it can be inferred that the weld has superior UTS and YS than BMs and it is only because of the superior deformation resistance of the Ni-based ERNiCrCoMo-1 weld not only at room temperature but also at high temperature. The gauge area of the specimen includes the weld metal, HAZ and BM and each region has a distinct microstructure and mechanical properties. Hence, the deformation of each zone during tensile loading will also not be uniform and it is mainly controlled by the precipitation strengthening and grain size. The Mo and Cr in ERNiCrCoMo-1 filler contributes to the solution strengthening and precipitation strengthening by the evolution of the secondary phases (Mo_6_C, M_23_C_6_). The Mo-rich phases also contributes to the hardening effect that reflects in RT and HT test results. These phases are more effective in resisting plastic deformation and raising the stress level during tensile loading by blocking and impeding the passage of dislocations. That one could be the possible cause of the failure of the test specimen from weaker P92 BM rather than weld metal. The UTS was 278 MPa for NVG-HT and 275 MPa for DVG-HT (Table 3). Because of the failure from P92 BM during the HT test, a P92 BM sample was also tested at a high temperature, i.e. 650 °C (P92BM-HT). The purpose of the P92BM-HT test was to compare the properties of P92 BM and welded joints at high-temperature. From the results, it was concluded that due to the failure of the welded joint from P92 BM in the HT test, UTS was also close to P92BM-HT (UTS: 318 ± 2 MPa). The fracture surface of the P92BM-HT, NVG-HT and DVG-HT is characterized further and presented in Fig. 26d–f. Figure 26d,e shows the macro-profiles of the fractured tip of the ruptured HT specimen (mentioned in corner of each image). The average diameter at the fracture frontier was 1.55 mm and 1.60 mm for NVG-HT and DVG-HT, respectively while for P92BM-HT it was 1.29 mm. The macrograph (Fig. 26b) and top view of the fracture surface (Fig. 26d–f) show a cup-cone-shaped profile. From macro-profiles of both NVG-HT and DVG-HT specimens, dimples can be observed clearly. The cup-cone formation with necking and the presence of higher-density dimples at the fracture surface is a characteristic of ductile fracture. The fracture surface of both NVG-HT and DVG-HT exhibits a higher density of dimples and microvoids. However, dimples and voids are observed bigger in size for DVG-HT (Fig. 26e,f).

### Residual stresses

**Figure 27:**
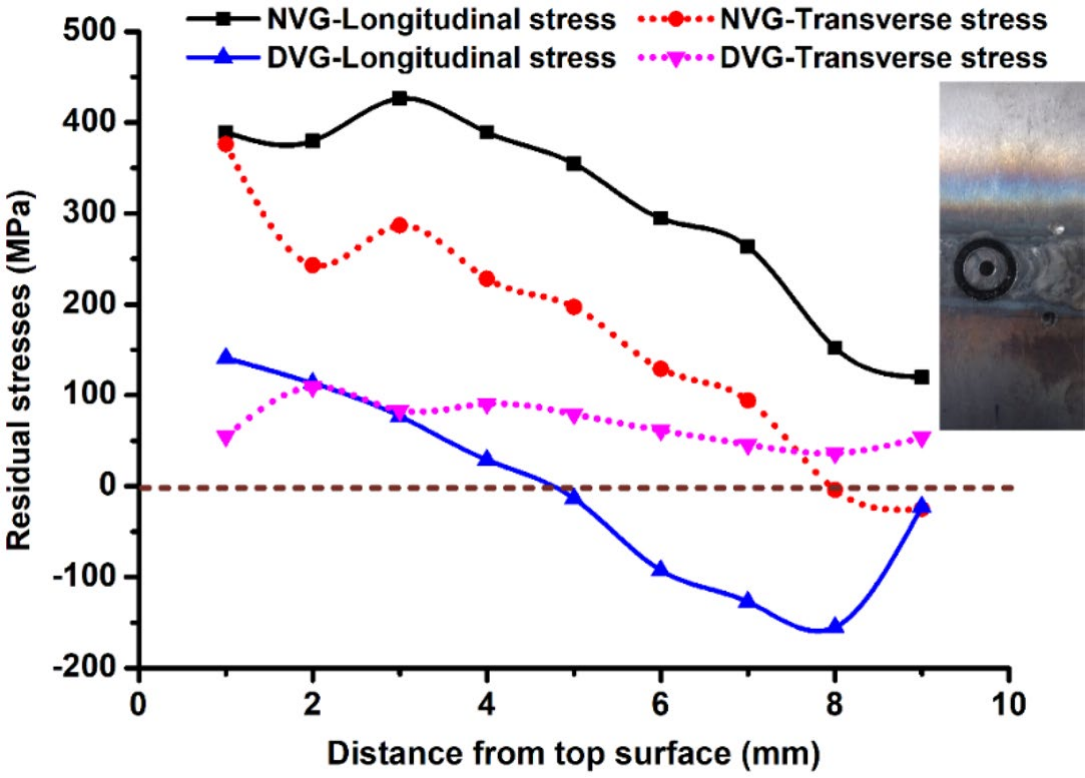
Residual stress plot for DVG and NVG welds joint.

The trend of residual stress variation along the thickness of the welded plate is given in Fig. 27. In the NVG joint, the longitudinal stresses are found to be tensile in nature throughout the thickness. The maximum and minimum magnitude of longitudinal stress was 426 MPa and 120 MPa, respectively which were measured at depth of 3 mm (filling pass/capping pass region) and 9 mm (root region), respectively from the top surface. The nature of transverse stress was tensile in nature up to a depth of 7 mm from the top surface and then becomes compressive. The maximum and minimum magnitude of transverse stress were 376 MPa and − 25 MPa, respectively which were measured at depth of 1 mm (capping pass region) and 9 mm (root region), respectively from the top surface. The trend of longitudinal stress was observed to be similar in nature for both NVG and DVG welds joints. However, the magnitude of the stresses was measured lower in DVG welds joint. The nature of the longitudinal stress in DVG was tensile up to the depth of 4 mm and beyond that, it showed compressive nature. The peak magnitude of the stress in the DVG joint was 141 MPa at the top surface which showed a reduction of 202% as compared to the peak magnitude of the stress in the NVG joint. In compressive nature, the peak magnitude of 155 MPa was observed. The nature of the stress was also compressive in the centre region of DVG this could be due to the reheating effect of the subsequent welding passes. The nature of transverse stress was observed to be tensile in nature throughout the plate thickness with a maximum and minimum magnitude of 109 MPa (2 mm from the top surface) and 36 MPa (2 mm from the bottom surface), respectively. As compared to NVG welds joint, DVG welds joint showed a reduction in peak transverse stress value by 245%.

## Conclusions

1. The SEM/EDS, optical, and EPMA mapping were conducted on the interface between ERNiCrCoMo-1 weld and P92 steel. An uneven distribution of beach, peninsula and island structures along the fusion boundary of P92 steel was confirmed. The diffusion of the Fe from P92 steel to ERNiCrCoMo-1 weld and Cr, Co, Mo, and Ni from ERNiCrCoMo-1 weld to P92 steel were also observed. Due to the closeness in composition in the melting point of Alloy 617 and ERNiCrCoMo-1 weld, a negligible diffusion is observed at the interface of Alloy 617. The beach, i.e. unmixed zone, was also not detected at the interface of Alloy 617, which confirmed the higher dilution.
2. The phase study was carried out using SEM/EDS, EPMA, and XRD. Alloy 617 BM had the major precipitates of M_23_C_6_, M_6_C, Ti(C, N), Ni_3_Ti, Ni–Cr–Fe and Ni–Cr–Co–Mo, while in P92 BM, the major precipitates were M_23_C_6_, M_7_C_3_ and (V, Nb)(C, N). The major phase of Mo-rich Mo_6_C and Cr-rich M_23_C_6_ were confirmed in ERNiCrCoMo-1 weld using EPMA and SEM/EDS. In the terminal stage of solidification, Mo leaves the dendrite core, is rejected to inter-dendritic regions, and enriches the region with Mo-rich phases like Mo_6_C. The other phases in ERNiCrCoMo-1 weld were Ni_3_(Al, Ti), Ti(C, N), Cr_7_C_3_ and Mo_2_C, which were confirmed from XRD analysis.
3. The weld metal showed the fully austenitic microstructure containing distinct dendrites. The variation in ERNiCrCoMo-1 weld microstructure in respect of composition and dendritic structure is observed from top to root as well as in a transverse direction.
4. Grain growth in HAZ of P92 and Alloy 617 was witnessed from the optical image. The precipitate dissolution and coarsening are other features which were observed in HAZ. A significant effect of grain coarsening and precipitates dissolution on impact and hardness properties of Alloy 617 and P92 HAZ were confirmed from the hardness and impact test results.
5. The impact energy of the ERNiCrCoMo-1 weld was 99 ± 4 J for the NVG welds joint and 91 ± 3 J for the DVG welds joint, which was lower than the BMs. However, welded joint met the criteria of boiler application (minimum 42 J as per European Standard EN ISO15614-1:2017 and 80 J as per fast breeder reactor application) in respect of impact energy. The Mo-rich phases introduced in ERNiCrCoMo-1 weld impart the hardening to the weld metal that reflected in hardness results however, during impact loading, it facilitates the crack nucleation due to high-stress intensity and considers as one possible case of poor impact energy value of ERNiCrCoMo-1 weld than BMs. The presence of this phase was also witnessed from the EDS results of the particles present at the fracture surface.
6. The hardness survey indicated the variation in longitudinal and transverse directions. The variation in ERNiCrCoMo-1 weld hardness from top to root or from a centre region to region adjacent to the boundary could be attributed to variations in their dendritic microstructure and chemical composition. The composition gradient between the dendrite core and inter-dendritic areas also imparted the hardness variation in the weld. The maximum and minimum hardness was measured in P92 CGHAZ and ICHAZ, respectively. The hardness of the unmixed zone also varied from top to bottom, and it was between 238 and 277 HV at the P92 interface for NVG and 229–279 HV for DVG welds joint. The peak hardness in weld metal was measured corresponding to the root pass and it could be due to the higher density of phases and equiaxed microstructure that arose due to the reheating effect of subsequent passes.
7. The hardening effect of the Mo-rich phases was reflected clearly in tensile test results. The NVG and DVG weld joint samples failed at P92 BM in both room and high-temperature tensile testing. The UTS was 748 + 2 MPa for NVG-RT and 696 ± 5 MPa for DVG-RT. The UTS was 278 MPa for NVG-HT and 275 MPa for DVG-HT.
8. The groove design has seen a negligible effect on the microstructure characteristic and hardness/impact/tensile properties. However, distortion and residual stresses in NVG welds joint were measured higher than in DVG welds joint. The maximum and minimum residual stresses were 426 MPa and 120 MPa for NVG welds. The peak magnitude of the longitudinal stress in the DVG joint was 141 MPa at the top surface, showing a reduction of 202% compared to the peak magnitude of the stress in the NVG joint.

## Data Availability

The datasets used and/or analysed during the current study available from the corresponding author on reasonable request.
